# Proteomics-Based Transporter Identification by the PICK Method: Involvement of TM7SF3 and LHFPL6 in Proton-Coupled Organic Cation Antiport at the Blood–Brain Barrier

**DOI:** 10.3390/pharmaceutics14081683

**Published:** 2022-08-12

**Authors:** Toshiki Kurosawa, Yuma Tega, Yasuo Uchida, Kei Higuchi, Hidetsugu Tabata, Takaaki Sumiyoshi, Yoshiyuki Kubo, Tetsuya Terasaki, Yoshiharu Deguchi

**Affiliations:** 1 Laboratory of Drug Disposition and Pharmacokinetics, Faculty of Pharma-Sciences, Teikyo University, Tokyo 173-8605, Japan; 2 Department of Pharmaceutical Sciences, College of Pharmacy, University of Kentucky, Lexington, KY 40536, USA; 3 Division of Membrane Transport and Drug Targeting, Graduate School of Pharmaceutical Sciences, Tohoku University, Sendai 980-8578, Japan; 4 Department of Biopharmaceutics, School of Pharmacy, Tokyo University of Pharmacy and Life Sciences, Tokyo 192-0392, Japan; 5 Laboratory of Medicinal Chemistry, Faculty of Pharma-Sciences, Teikyo University, Tokyo 173-8605, Japan; 6 Department of Life Science and Biotechnology, Faculty of Chemistry, Materials and Bioengineering, Kansai University, Osaka 564-8680, Japan

**Keywords:** proton-coupled organic cation antiporter, blood–brain barrier, photo-affinity labeling, proteomics, SWATH-MS (sequential window acquisition of all theoretical-mass spectra)

## Abstract

A proton-coupled organic cation (H^+^/OC) antiporter working at the blood–brain barrier (BBB) in humans and rodents is thought to be a promising candidate for the efficient delivery of cationic drugs to the brain. Therefore, it is important to identify the molecular entity that exhibits this activity. Here, for this purpose, we established the **P**roteomics-based **I**dentification of transporter by **C**rosslinking substrate in **K**eyhole (PICK) method, which combines photo-affinity labeling with comprehensive proteomics analysis using SWATH-MS. Using preselected criteria, the PICK method generated sixteen candidate proteins. From these, knockdown screening in hCMEC/D3 cells, an in vitro BBB model, identified two proteins, TM7SF3 and LHFPL6, as candidates for the H^+^/OC antiporter. We synthesized a novel H^+^/OC antiporter substrate for functional analysis of TM7SF3 and LHFPL6 in hCMEC/D3 cells and HEK293 cells. The results suggested that both TM7SF3 and LHFPL6 are components of the H^+^/OC antiporter.

## 1. Introduction

The pyrilamine (PYR)-sensitive proton-coupled organic cation (H^+^/OC) antiporter working at the blood–brain barrier (BBB) in humans and rodents is thought to be a promising candidate for drug delivery, since various psychotropic drugs have cationic forms at physiological pH, and carrier-mediated transport of such drugs across the BBB is inhibited by lipophilic cationic drugs, such as diphenhydramine (DPH) and imipramine [1]. Furthermore, in vivo brain microdialysis studies of DPH and oxycodone (OXY) revealed three- to five-fold higher unbound concentrations in the brain interstitial fluid (ISF) than in blood [2,3]. A similar influx transport system was found in an in situ mouse brain perfusion study of nicotine and clonidine (CLO) [4,5], as well as an in vivo study on the transport of cationic drugs at the blood–retinal barrier (BRB) and in the liver [6,7]. Possible involvement of a proton-coupled organic cation (H^+^/OC) antiporter was supported by studies using hCMEC/D3 cells, an in vitro model cell line of human BBB, as well as conditionally immortalized capillary endothelial cells of rat brain and retina [8,9,10]. The pharmacological significance of the H^+^/OC antiporter was also supported by a pharmacophore model study designed to predict inhibitors [11]. Interestingly, hybrid molecules constructed by combining a histone deacetylase (HDAC) inhibitor or non-steroidal anti-inflammatory drug (NSAID) with an H^+^/OC antiporter substrate showed increased blood–brain barrier (BBB) permeability [12,13].

Thus, although the molecular identity of the H^+^/OC antiporter at the BBB remains un-clear, it appears to be a promising candidate for the efficient delivery of central nervous system (CNS)-active drugs into the brain [9,14,15,16,17]. Various organic cation transporters, including organic cation transporters (OCTs), organic cation/carnitine transporters (OCTNs) and multidrug and toxin extrusion proteins (MATEs), have been identified, molecularly cloned and characterized [18], but none of these molecules has transport properties consistent with those of the H^+^/OC antiporter. The molecular nature of the H^+^/OC antiporter at the BBB has remained elusive for at least 30 years. A possible explanation for this would be that the H^+^/OC antiporter is a protein complex, not a single protein, and thus might not be amenable to identification by conventional methods such as the gene level approach with loss or gain of function.

**Scheme 1:**
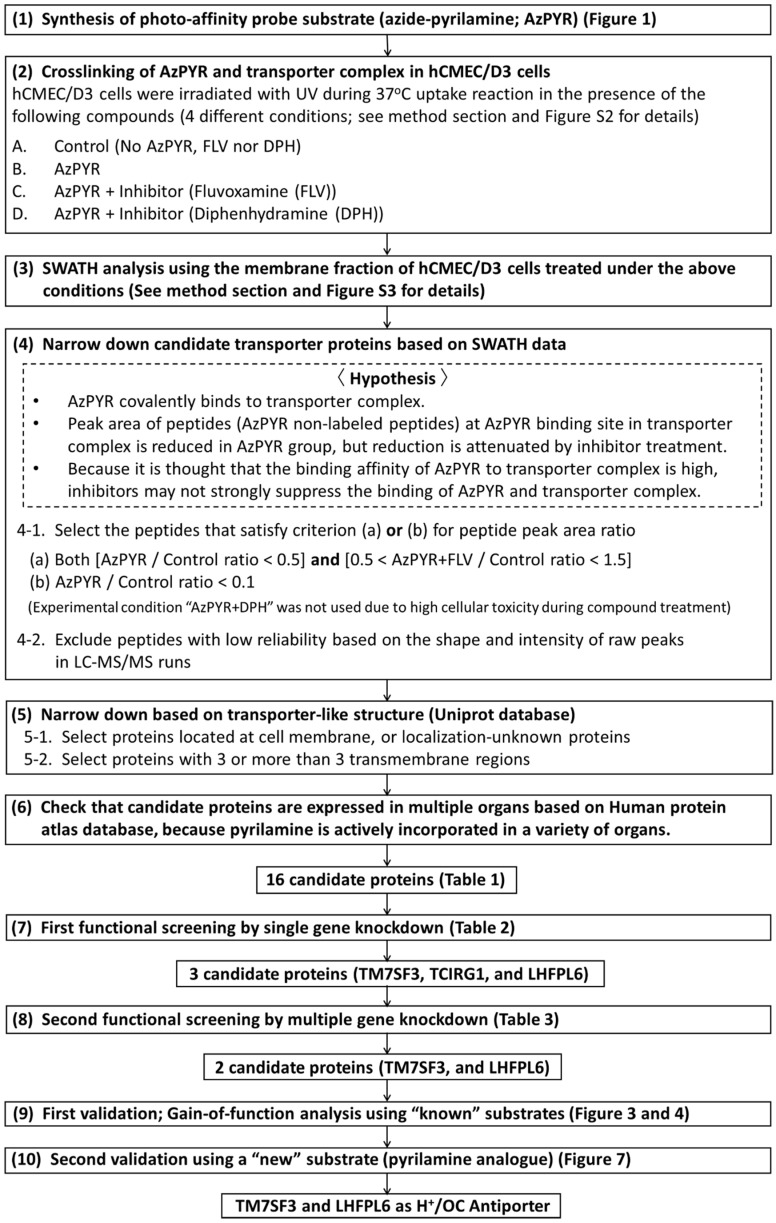
Strategy for identification of transporter complex by PICK (**P**roteomics-based **I**dentification of transporter by **C**rosslinking substrate in **K**eyhole) method.

Since 2000, proteomics technology has been developed and used in a variety of scientific fields [19], including the identification of receptors by the application of sophisticated crosslinkers [20]. The application of this crosslinking-based proteomics approach to substrate–transporter interactions have the potential to efficiently identify multiple constituent molecules of the transporter complex. The photoreactive azide group (one of the crosslinkers) is small, and therefore, azide modification to the substrate of transporter would not significantly change the structure of the substrate, making it easier to enter the transporter’s binding site. Hence, a strategy that combines the use of azide-modified substrates and proteomic techniques with excellent coverage and accuracy would be useful for identification of the H^+^/OC antiporter. We have devised a transporter identification method based on the strategy in Scheme 1 and named it the “PICK” (**P**roteomics-based **I**dentification of transporter by **C**rosslinking substrate in **K**eyhole) method. This combines the use of azide-modified transporter substrates with a proteomic technique that affords excellent coverage and accuracy, and also utilizes inhibitors to increase the specificity/accuracy of transporter identification.

In the present study, the PICK method was applied for the molecular identification of the H^+^/OC antiporter, using azide-pyrilamine (AzPYR) as a photo-affinity probe [21,22]. Candidate proteins selected by the PICK method were taken forward for functional studies utilizing single and multiple gene knockdown analyses. For final confirmation, we used cell lines stably expressing the putative transporter components, together with a newly synthesized H^+^/OC antiporter substrate, which was confirmed to have appropriate membrane permeability.

## 2. Materials and Methods

### 2.1. Reagents

Reagents used in this study were purchased from Fujifilm Wako Pure Chemical Industries (Osaka, Japan) and Sigma-Aldrich Company (St. Louis, MO, USA), unless otherwise specified. Varenicline tartrate and fluvoxamine maleate were purchased from Abcam (Cambridge, MA, USA) and Tokyo Chemical Industry (Tokyo, Japan), respectively.

### 2.2. Cell Culture

hCMEC/D3 cells were kindly provided by Dr. Pierre-Oliver Couraud (Institut Cochin, Paris, France) under license from INSERM and cultured on rat collagen type I-coated dishes in EBM-2 medium (Lonza, Basel, Switzerland). HEK293 cells were cultured on poly-D-lysine-coated dishes in Dulbecco’s modified Eagle’s medium (D-MEM, high glucose; Thermo Fisher Scientific, Waltham, MA, USA) supplemented with 10% fetal bovine serum, 1% penicillin-streptomycin and NaHCO_3_ (final concentration; 1.5 g/L). These cells were maintained in an atmosphere of 95% air and 5% CO2. For details of cell culture conditions and medium composition, see the Supplementary Materials.

### 2.3. Uptake Study

Uptake studies with hCMEC/D3 cells and HEK293 cells were performed in accordance with previous reports [16,17]. Briefly, we seeded hCMEC/D3 cells in collagen I-coated 24-well plates (BD Biosciences, Franklin Lakes, NJ, USA) and HEK293 cells in poly-D-lysine (Thermo Fisher Scientific)-coated 24-well plates at a density of 4.0 × 10^4^ cells/cm^2^. Uptake studies were performed after 3 days. The cells were washed in uptake buffer and preincubated in fresh uptake buffer for 20 min at 37 °C, and then uptake of the compound was measured at the designated times. Uptake in each experiment was evaluated by calculating the cell-to-medium (C/M) ratio (μL/mg protein). The amount of protein in each well was determined with a Micro BCA protein assay kit (Thermo Fisher Scientific). The drugs and compounds were quantified by an LC-MS/MS system consisting of a Nexera XR HPLC system (Shimazu, Kyoto, Japan) and a Qtrap 4500 (AB Sciex, Foster City, CA, USA) mass spectrometer with an electrospray ionization interface in positive ion mode. Details of the measurement methods and ionization conditions for each drug and compound are given in the Supplementary Materials.

### 2.4. Design, Synthesis and Evaluation of Photo-Affinity Probe (Azide-Pyrilamine; AzPYR)

**Figure 1:**
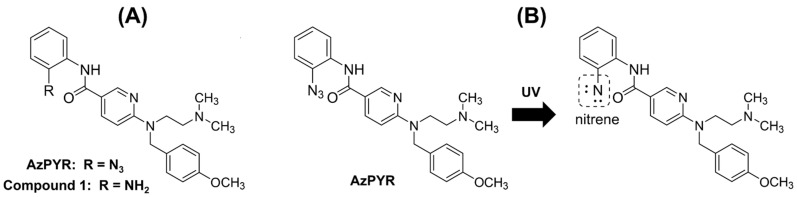
(**A**) Chemical structures of AzPYR and compound **1**. (**B**) Production of the phenyl nitrene moiety, which forms a covalent bond with the H^+^/OC antiporter.

In our program to identify the H^+^/OC antiporter, we designed AzPYR as a photo-affinity probe (Figure 1A). Based on the idea that histone deacetylase inhibitor **1** (Figure 1A) is a relatively slow-reacting H^+^/OC antiporter substrate [12], we introduced an azide moiety at the phenyl group of compound **1**. UV irradiation (<300 nm) of AzPYR generated an active phenyl nitrene that binds covalently with the H^+^/OC antiporter (Figure 1B). According to the reported procedure for converting an aniline moiety to phenyl azide [23], we synthesized AzPYR from compound **1** using 2-azido-1,3-dimethylimidazolinium hexafluorophosphate (Figure S1, see Supplementary Materials).

To test the binding ability of AzPYR to the H^+^/OC antiporter, we examined the inhibitory effect of AzPYR on the antiporter-mediated uptake activity. The uptakes of DPH (at 1 μM) and PYR (at 5 μM) by hCMEC/D3 cells for 0.5 min were measured at 37 °C in the presence or absence of 0.4 mM AzPYR. For the photo-cross-linking reaction, the cells were pretreated with AzPYR under UV light for 5 min at 37 °C. Unreacted AzPYR was removed by incubation in the buffer.

### 2.5. Crosslinking of AzPYR and Transporter Complex in hCMEC/D3 Cells

hCMEC/D3 cells were seeded on plastic dishes. After reaching confluence, the cells were preincubated in the buffer with or without an H^+^/OC antiporter inhibitor (fluvoxamine (FLV) or DPH). The cells were treated with 0.1 mM AzPYR in the presence and absence of inhibitor (0.5 mM FLV or 0.5 mM DPH) for 5 min at 37 °C under UV light (302 nm) generated by a benchtop trans-illuminator (Analytik Jena AG, Jena, Germany), then washed twice with ice-cold buffer and transferred into PBS (-) with a cell scraper. Cells irradiated with UV in the absence of AzPYR and inhibitor were also collected as a control. The cell suspensions were centrifuged at 1000× *g* for 5 min at 4 °C, and the supernatants were removed. The cell pellets were stored at −80 °C until SWATH-MS (Sequential Window Acquisition of all Theoretical fragment ion spectra-Mass Spectra) analysis. Photoaffinity labeling of the H^+^/OC antiporter using hCMEC/D3 cells is illustrated schematically in the Supplementary Materials (Figure S2).

### 2.6. SWATH Analysis to “PICK” Candidate Transporter Proteins

The membrane fractions were isolated from the hCMEC/D3 cells treated as described above by using a Minute Plasma Membrane Protein Isolation Kit^®^ (Invent Biotechnologies). Trypsin digestion of membrane fractions and C18 clean-up were conducted as described previously [24]. The cleaned peptide samples were injected into a NanoLC 425 system (Eksigent Technologies, Dublin, CA, USA) coupled with an electrospray-ionization Triple TOF 5600 mass spectrometer (SCIEX, Framingham, MA, USA) set up for a single direct injection. SWATH-MS data were acquired as previously described [25]. Data extraction from the SWATH chromatogram were processed using the SWATH Processing Micro App in Peakview (SCIEX) with a 10% false discovery rate threshold as previously described [26]. Unreliable peaks and peptides were removed as described [27]. Briefly, transitions with a peak area of more than 1000 counts in the control group were extracted. Transitions whose peak area was >10-fold different between two replicates were removed. Then, peptides with only one or two transitions were removed. Furthermore, nonspecific and unreliable peptides were removed by applying in silico peptide selection criteria [27]. For the remaining peptides, the peak areas at the peptide level were calculated as the average values after normalizing differences in signal intensity between the different transitions. Subsequently, the candidate transporter proteins were narrowed down according to steps 4, 5, and 6 shown in the flowchart (Scheme 1). The detailed workflow of the SWATH analysis is shown in the Supplementary Materials (Figure S3).

### 2.7. Functional Screening by Single or Multiple Gene Knockdown

hCMEC/D3 cells were seeded on 24-well plates at a density of 2.75 × 10^4^ cells/well on the day before siRNA treatment. They were incubated in Opti-MEM I reduced serum medium (Thermo Fisher Scientific, Waltham, MA, USA) including Lipofectamine RNAi MAX (Millipore-Sigma, Burlington, NJ, USA) and two kinds of siRNA for one target gene, each at a final concentration of 5 nM, for 24 h. Then, the medium was changed to EBM-2 medium, and the cells were cultured for a further 48 h without siRNA. As a control group, the cells were incubated with the same amount of negative control siRNA (Qiagen, Venlo, The Netherlands) instead of target-specific siRNA. Product information for targeted siRNA and negative control siRNA is provided in the Supplementary Materials (Table S2).

To confirm knockdown of target genes, total RNA extraction from hCMEC/D3 cells was performed using NucleoSpin RNA Plus (Macherey-Nagel, Düren, Germany) according to the supplied manual. Reverse transcription reactions from total RNA to cDNA were performed with the combination of SuperScript III Reverse Transcriptase (Thermo Fisher Scientific) and Ribonuclease Inhibitor (TaKaRa Biomedicals, Shiga, Japan). Briefly, 1 μg of total RNA was mixed with 250 ng of random primer and the supplied dNTPs and incubated at 65 °C for 5 min. The mixture was then mixed with a defined volume of SuperScript III and Ribonuclease Inhibitor and incubated at 50 °C for 60 min and 70 °C for 15 min. PCR was performed using a mixture of 10 ng of cDNA, 5 pmol of sense/antisense primers and SYBR Select Master Mix (Thermo Fisher Scientific) on a 7500 Fast Real-Time PCR System (Applied Biosystems) according to the following thermocycling program: 1 cycle of holding stage at 50 °C for 2 min and 95 °C for 2 min, 40 cycles of PCR reaction stage at 95 °C for 3 s and 60 °C for 0.5 min, and 1 cycle of melt curve stage at 95 °C for 0.25 min, 60 °C for 1 min and 95 °C for 0.5 min. Primer sequences are listed in Supplementary Materials (Table S3). Relative mRNA expression of each target protein was calculated by the ΔCt method, corrected for the mRNA expression of TATA-binding protein (TBP; a house-keeping gene).

### 2.8. First Validation; Gain-of-Function Analysis Using “Known” Substrates

HEK293 cells expressing transmembrane 7 superfamily member 3 (TM7SF3) and/or LHFPL tetraspan subfamily member 6 protein (LHFPL6) were generated to assess the involvement of these molecules in the transport of H^+^/OC antiporter substrates. pcDNA TM3.1 (+) vector and pcDNA TM3.1/Zeo (+) vector containing the coding region of TM7SF3 (GenBank accession number: NM_016551.3) or LHFPL6 (GenBank accession number: NM_005780.3), respectively, were purchased from Genscript (Piscataway, NJ, USA). The cells were incubated in Opti-MEM I reduced serum medium (Thermo Fisher Scientific) with the vector and Lipofectamine 3000 (Thermo Fisher Scientific) for 6 h. The medium was subsequently changed to D-MEM without antibiotics. For transient expression, the cells were incubated for a further 48 h and used for the uptake experiment. To obtain stably expressing cells, incubation was continued for another 24 h, and then the cells were cultured in the medium with antibiotics (400 μg/mL Zeocin and G418) to obtain resistant cells. These were proliferated, cloned and used for uptake experiments. The mRNA expression of TM7SF3 and LHFPL6 in HEK293 cells was measured by the quantitative PCR method described above.

### 2.9. Second Validation Using a “New” Substrate (Pyrilamine Analogue)

Commercially available 4-bromonicotinate (**1**) was treated with *N,N*-dimethylethylendiamine in pyridine to obtain the secondary amine (**2**) [28,29]. Benzyl derivative (**3**) [12] was obtained by *N*-alkylation of **2** with 4-methoxybenzyl chloride, then hydrolyzed and amidated with ethyl 4-aminobutyrate to obtain **4**. The methods and the properties of each compound are given in the Supplementary Materials (Figure S4). The uptake mechanism of the pyrilamine analogue was analyzed by uptake study in hCMEC/D3 cells. To examine the effects of sodium ions and membrane potential on the uptake, NaCl in the uptake buffer was replaced with LiCl/cholineCl or KCl, respectively. Furthermore, the effect of metabolic energy on uptake was evaluated by replacing glucose in the uptake buffer with non-metabolizable 3-*O*-methyl-glucose and adding 0.1% NaN_3_. The influence of intracellular pH was evaluated by adding 30 mM NH_4_Cl to the uptake buffer. For acidification of intracellular pH, NH_4_Cl was included from the preincubation stage; for alkalinization, NH_4_Cl was added simultaneously with the pyrilamine analogue. The composition of the uptake buffer in each experiment is given in the Supplementary Materials. To evaluate the inhibition profile of the pyrilamine analogue in hCMEC/D3 cells, various inhibitors (1 mM) were added simultaneously with the pyrilamine analogue. The inhibitors used were PYR, DPH, CLO, memantine (MEM), varenicline (VAR), tramadol (TRA), naltrexone (NAL) (a substrate and inhibitor of the H^+^/OC antiporter), 1-methyl-4-phenylpyridinium (MPP^+^, a substrate and inhibitor of OCTs and plasma membrane monoamine transporter (PMAT)), p-aminohippuric acid (PAH, a substrate and inhibitor of OATs and organic anion transporting polypeptides (OATPs)), tetraethylammonium (TEA, a substrate and inhibitor of OCTs and MATE1) and L-carnitine (a substrate of OCTN2). In order to calculate kinetic parameters in hCMEC/D3 cells, the uptake of pyrilamine analogue was analyzed by preparing Michaelis–Menten plots based on the following equation (parameters are defined in the Supplementary Materials).

*V* = (*V*_max_ × *S*)/(*K*_m_ + *S*) + (*K*_d_ × *S*) (1)

The involvement of TM7SF3 and LHFPL6 in the uptake of the pyrilamine analogue was evaluated using knockdown hCMEC/D3 cells and TM7SF3- and/or LHFPL6-expressing HEK293 cells, generated as described above.

### 2.10. Statistical Analysis

All values are presented as the mean ± standard error. Statistical analysis of the data was performed with Student’s *t*-test and one-way ANOVA followed by Dunnett’s test for single and multiple comparisons, respectively. Values of *p* < 0.05 and 0.01 were considered to represent statistically significant differences. Unless otherwise specified in the figure legend, a significant difference analysis with the Dunnett’s test was performed.

## 3. Results

### 3.1. Binding of AzPYR to H^+^/OC Antiporter

**Figure 2:**
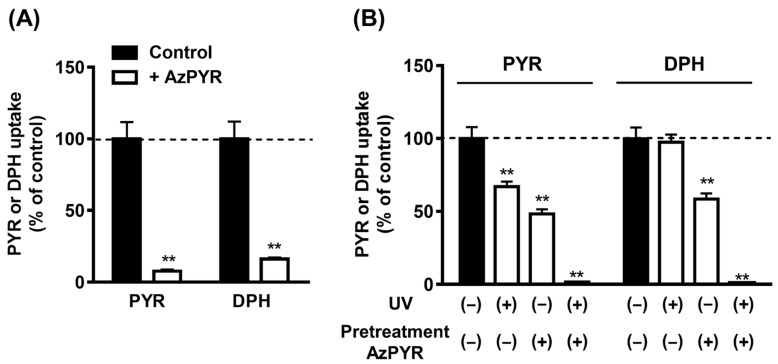
(**A**) Inhibitory effect of AzPYR on H^+^/OC antiporter-mediated uptake. The uptakes of PYR and DPH by hCMEC/D3 cells were measured in presence or absence of AzPYR at 0.4 mM under room light. (**B**) Effect of photo-cross-linking reaction on H^+^/OC antiporter activity. hCMEC/D3 cells were pretreated in the buffer with or without AzPYR under UV light or room light for 5 min at 37 °C. The treated cells were washed and further incubated in buffer to remove the unreacted AzPYR, and then the uptakes of PYR and DPH were measured. Each point represents the mean ± standard error (*n* = 3–4). ** *p* < 0.01 compared to the corresponding control uptake or non-treatment uptake. Significant difference in Figure 2A was examined with *t*-test.

The inhibitory effects of AzPYR on H^+^/OC antiporter-mediated uptakes of PYR and DPH, which are representative substrates, was examined using hCMEC/D3 cells in order to confirm the binding of AzPYR to the antiporter. AzPYR at 0.4 mM significantly reduced the uptakes of PYR and DPH (Figure 2A). Pretreatment of hCMEC/D3 cells with AzPYR under UV light caused a significant and irreversible reduction in the uptakes of PYR and DPH, whereas pretreatment under room light caused a moderate and reversible reduction (Figure 2B). These results suggest that the synthesized AzPYR binds to the H^+^/OC antiporter and efficiently photo-labels it.

### 3.2. SWATH-Based Screening of H^+^/OC Antiporter

Our strategy for the identification of the H^+^/OC antiporter is shown in Scheme 1. We assumed that AzPYR covalently binds to the substrate-binding site of the H^+^/OC antiporter upon UV irradiation, and that inhibitors suppress AzPYR binding to the transporter. To distinguish the specific binding of AzPYR to the antiporter, we used DPH and FLV, which have been reported to be the inhibitors of the antiporter [30,31]. However, we finally abandoned the use of DPH because the DPH treatment at 1 mM resulted in lower attachment of hCMEC/D3 cells to the dishes under the conditions used for UV irradiation. In SWATH analysis, membrane fractions are digested with trypsin and measured by LC-MS/MS, so the individual tryptic peptides are separately quantified. We assumed that if the AzPYR/control ratio was smaller than 0.5 and the (AzPYR+FLV)/control ratio was in the range 0.5 to 1.5, the peptide was derived from a candidate transporter (Scheme 1). Furthermore, a peptide with an AzPYR/control ratio < 0.1 (regardless of the value of the (AzPYR+FLV)/control ratio) was also assumed to be derived from a candidate transporter, because if the affinity of AzPYR for the transporter was high, binding may not be strongly suppressed by the inhibitor (Scheme 1). Among the proteins meeting these criteria, we selected 16 that (1) are expressed at the cell membrane; (2) contain 3 or more transmembrane regions; (3) are expressed in multiple organs (Table 1). 

For LHFPL tetraspan subfamily member 6 protein, PRA1 family protein 3, solute carrier family 43 member 3 (SLC43A3), CD63 antigen, and aquaporin-3, the AzPYR/control ratio was smaller than 0.1. The other 11 proteins were selected as molecules with AzPYR/control ratio < 0.5 and 0.5 < (AzPYR+FLV)/control ratio < 1.5 (Table 1). Note that the value of “AzPYR+DPH” was not used as a criterion due to low cell attachment during DPH treatment. However, as reference data, the values of (AzPYR+DPH)/control ratios are also listed for these 16 proteins in Table 1.

### 3.3. First Functional Screening by Single Gene Knockdown

As a first functional screening, we assessed the effect of single gene knockdown on the uptake of H^+^/OC antiporter substrates and a non-substrate, gabapentin, by hCMEC/D3 cells. The mRNA reductions resulting from siRNA treatment are shown in Figure S5. All targeted mRNA levels were decreased by more than 71% except for SLC12A5, SLC43A3, and aquaporin-3. Further, TCIRG1 knockdown decreased PYR, TRA, and DPH uptakes by 50, 43, and 16%, respectively (Table 2), although the differences were not statistically significant. In addition, TM7SF3 and LHFPL6 knockdown reduced the uptakes of H^+^/OC antiporter substrates by more than 30% and 25%, respectively. On the other hand, these gene knockdowns did not decrease gabapentin uptake, suggesting that the effect is specific to H^+^/OC antiporter substrates. CD9 knockdown decreased the PYR, TRA, and DPH uptakes, but the cell morphology was markedly changed (Figure S6). Hence, we focused on TCIRG1, TM7SF3, and LHFPL6 as candidate molecules.

### 3.4. Second Functional Screening by Multiple Gene Knockdown

We postulated that the H^+^/OC antiporter might be composed of more than one protein. Therefore, we assessed the effect of multiple gene knockdown on the uptake of H^+^/OC antiporter substrates by hCMEC/D3 cells as a second screening (Table 3). As shown in Figure S5, mRNA levels were decreased by more than 60% in the siRNA-treated groups. TCIRG1 and LHFPL6 knockdown decreased OXY and VAR uptakes by 25 and 39%, respectively, but had little effect on PYR and TRA uptakes. TCIRG1 and TM7SF3 siRNA treatment had an effect similar to that of TCIRG1 and LHFPL6 knockdown. However, TM7SF3 and LHFPL6 siRNA treatment resulted in an over 31% decrease in the uptakes of all H^+^/OC antiporter substrates, suggesting that the combination of TM7SF3 and LHFPL6 reduction could be critical for H^+^/OC antiporter-mediated uptake.

### 3.5. First Validation; Gain-of-Function Analysis Using “Known” Substrates

**Figure 3:**
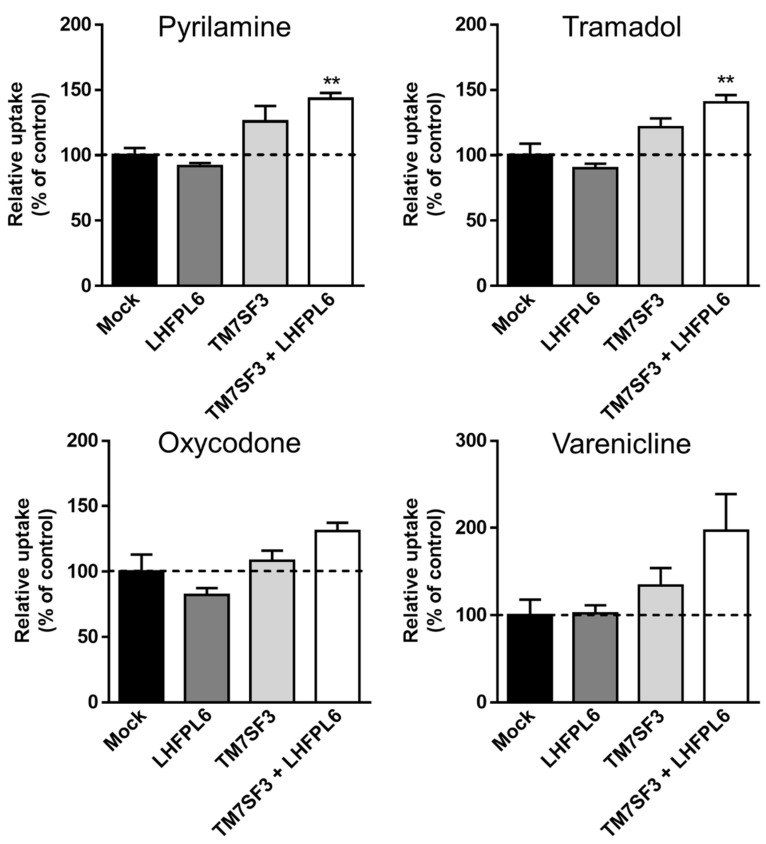
Uptakes of H^+^/OC antiporter substrates by HEK293 cells transiently transfected with LHFPL6 and/or TM7SF3. The uptake study was performed at 37 °C for 1 min in HEK293 cells transiently transfected with LHFPL6 and/or TM7SF3. Each column represents the mean ± standard error (*n* = 4). ** *p* < 0.01, significantly different from the uptake by mock cells.

**Figure 4:**
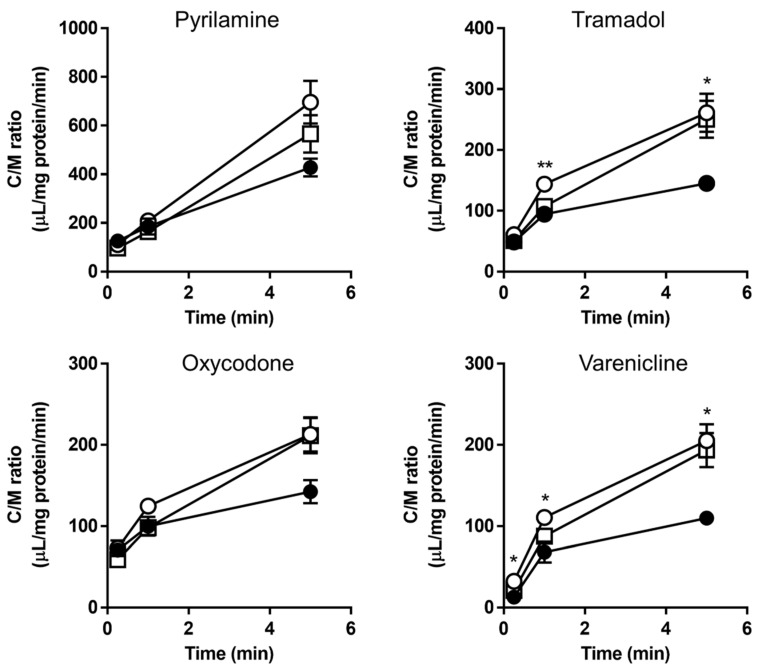
Uptake of H^+^/OC antiporter substrates by HEK293 cells stably double-transfected with LHFPL6 and TM7SF3. The time-courses of uptake were assessed at 37 °C in mock (closed circle) and HEK293 cells stably transfected with TM7SF3 (open circle) or both LHFPL6 and TM7SF3 (open square). Each point represents the mean ± standard error (*n* = 3). * *p* < 0.05 and ** *p* < 0.01 indicate significant differences in the uptakes by mock and TM7SF3-transfected cells.

For validation of the involvement of TM7SF3 and LHFPL6 in H^+^/OC antiporter-mediated uptake, we conducted a gain-of-function analysis with HEK293 cells. As shown in Figure 3, there was no difference in uptake of H^+^/OC antiporter substrates between mock and transiently LHFPL6-expressing cells. On the other hand, PYR, TRA, and VAR uptakes were increased by 21–34% in cells transiently expressing TM7SF3, although the differences were not statistically significant. In addition, cells overexpressing both TM7SF3 and LHFPL6 showed increases in PYR and TRA uptakes by 43 and 40%, respectively. Again, these were not statistically significant, but OXY and VAR uptakes were also increased by 31 and 97%, respectively. To further study the transport function, we generated stably expressing HEK293 cells. These cells showed a time-dependent increase in uptakes of H^+^/OC antiporter substrates (Figure 4). Stably TM7SF3-expressing cells showed significantly higher TRA uptakes at 1 and 5 min and VAR uptakes at 0.25, 1, and 5 min compared with mock cells. The uptake of H^+^/OC antiporter substrates by stably TM7SF3 and LHFPL6-expressing cells as well as by TM7SF3-expressing cells tended to be increased at 5 min compared to mock cells. In contrast to transiently expressing cells, the effect of TM7SF3 and LHFPL6 co-expression was not additive in the stably expressing cells. The uptake of antipyrine, a passive diffusion marker, was not altered in these cells (Figure S7).

### 3.6. Second Validation Using a “New” Substrate (Pyrilamine Analogue)

**Figure 5:**
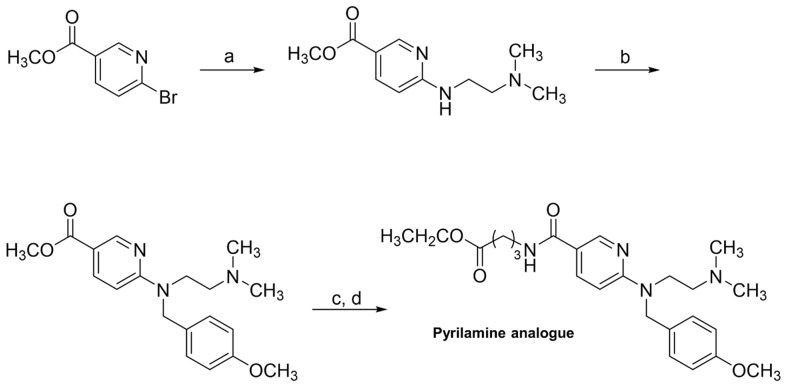
Synthesis of pyrilamine analogue as a new H^+^/OC antiporter substrate. (**a**) *N,N*-Dimethylethylendiamine, pyridine, 120 °C; (**b**) 4-methoxybenzyl chloride, NaH, THF rt; (**c**) aq. NaOH, CH_3_OH, 100 °C; (**d**) ethyl 4-aminobutyrate hydrochloride, ethyl-3-(3-dimethylaminopropyl) carbodiimide hydrochloride, 1-hydroxybenzotriazole, DIPEA, DMF, rt.

**Figure 6:**
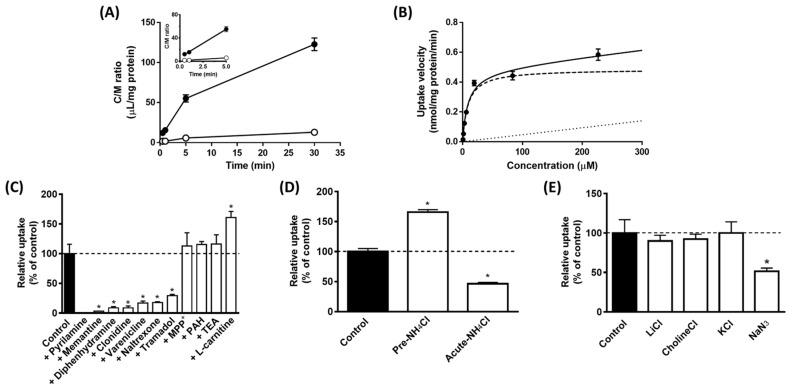
Transport characteristics of pyrilamine analogue in hCMEC/D3 cells. (**A**) Time- and temperature-dependent uptake experiments were performed at 37 °C (closed circles) and 4 °C (open circles) for up to 30 min. (**B**) The concentration-dependent uptake experiments were performed at 37 °C for 1 min (solid line). The dashed and dotted lines represent the saturable and non-saturable uptake components, respectively. (**C**) Inhibition experiments were performed at 37 °C for 1 min in the absence and presence of several inhibitors (1 mM). The black and white columns indicate inhibitory effects as percent of the control. (**D**,**E**) Effects of intracellular pH, extracellular Na^+^, membrane potential and metabolic energy on pyrilamine analogue uptake by hCMEC/D3 cells. Cellular uptake in the C-E is shown as percent of the control. Each value and column represent the mean ± standard error (*n* = 4). * *p* < 0.05 indicates a significant difference versus the control.

For second validation, we synthesized a new cell membrane-permeable substrate, a pyrilamine analogue, to further examine whether TM7SF3 and LHFPL6 are involved in the function of the H^+^/OC antiporter (Figure 5). We confirmed that the pyrilamine analogue is a substrate of the H^+^/OC antiporter in hCMEC/D3 cells. The C/M ratio of the pyrilamine analogue increased linearly from 0.5 to 5 min (Figure 6A), and the initial uptake rate (up to 5 min) was calculated to be 9.71 μL/mg protein/min. Furthermore, this uptake was reduced by approximately 90% at 4 °C. The uptake of pyrilamine analogue showed a *K*_m_ of 8.85 ± 2.15 μM and a *V*_max_ of 0.487 ± 0.052 nmol/mg protein/min for the saturable component and a *K*_d_ of 0.467 ± 0.260 μL/mg protein/min for the non-saturable component (Figure 6B). The uptake was significantly inhibited (to <16.7%) by PYR, MEM, DPH, CLO, VAR, NAL, and TRA, which are substrates and/or inhibitors of the H^+^/OC antiporter, but was not inhibited by MPP^+^, PAH, TEA, and L-carnitine, which are not substrates or inhibitors of the H^+^/OC antiporter (Figure 6C). The C/M ratio was increased to 165% or decreased to 46%, respectively, by intracellular acidification and alkalinization (Figure 6D). The uptake of the pyrilamine analogue was also reduced by NaN_3_, an energy-depleting agent, but was not affected by replacement of extracellular sodium ions with lithium, choline, or potassium ions (Figure 6E). Thus, the transport characteristics of the pyrilamine analogue are consistent with those of reported substrates of the H^+^/OC antiporter.

**Figure 7:**
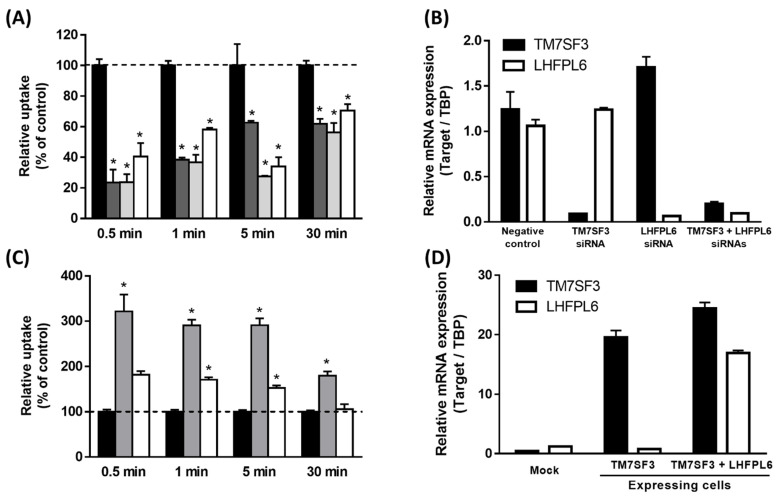
Effects of TM7SF3 and LHFPL6 on the cellular uptake of pyrilamine analogue. (**A**) Intracellular uptake of pyrilamine analogue in TM7SF3 or/and LHFPL6 knockdown hCMEC/D3 cells was measured at 37 °C, for 0.5, 1, 5 and 30 min. The black, dark gray, light gray and white columns represent the negative control, TM7SF3 knockdown, LHFPL6 knockdown, and their double knockdown cells, respectively. (**B**) The mRNA expression of TM7SF3 and LHFPL6 in hCMEC/D3 cells treated with the respective siRNAs. (**C**) Uptake of pyrilamine analogue in HEK293 cells stably expressing TM7SF3 or both TM7SF3 and LHFPL6 was evaluated at 37 °C. The black, gray, and white columns show the results for mock, TM7SF3-expressing, and TM7SF3 + LHFPL6-expressing cells, respectively. (**D**) The mRNA expression in HEK293 cells stably overexpressing TM7SF3 and LHFPL6 was measured by qPCR and normalized to that of TBP. Cellular uptake in A and C is shown as percent of the control. Each column represents the mean ± standard error (*n* = 4). * *p* < 0.05 indicates a significant difference versus the negative control.

To evaluate the contribution of TM7SF3 and LHFPL6 to the uptake of the pyrilamine analogue, we used hCMEC/D3 cells in which these genes were knocked down with siRNA. Transfection with siRNA blocked the expression of each of TM7SF3 and LHFPL6 by more than 80% (Figure 7B). Similar results were also obtained in the double knockdown of TM7SF3 and LHFPL6. The uptakes of pyrilamine analogue at 0.5 to 5 min were reduced by 23.6, 23.7 and 40.4% at 0.5 min, 38.5, 36.8 and 58.1% at 1 min and 62.7, 27.6 and 33.9% at 5 min in cells with knockdown of TM7SF3, LHFPL6, and TM7SF3 + LHFPL6, respectively, compared to the negative control (Figure 7A). The C/M ratio in these cells was significantly reduced at 30 min to 61.9%, 56.2%, and 70.5%, respectively, but the effects of knockdown declined time-dependently. In addition, the effects of gain-of-function were evaluated using HEK293 cells stably expressing TM7SF3 and LHFPL6 (Figure 4). The C/M ratio of the pyrilamine analogue at 0.5 min was increased to 321% and 181% in TM7SF3-expressing cells and TM7SF3 + LHFPL6-expressing cells, respectively (Figure 7C). Again, the increase in C/M ratio was attenuated with prolonged uptake time. The mRNA expression levels in these cells showed a more than 40-fold increase in TM7SF3 and a 15-fold increase in LHFPL6 (Figure 7D).

## 4. Discussion

The present study was designed to establish the molecular identity of the H^+^/OC antiporter at the BBB, using a combination of the PICK method and functional analyses with a cell-permeable pyrilamine analogue. The results indicated that both TM7SF3 and LHFPL6 are involved in the antiporter activity.

Screening of transporters using photoreactive azide compounds had been performed in the 1980s and 1990s. For example, this approach was used to study the rabbit small-intestinal Na^+^, D-glucose membrane transporter [32] and ATP transporter of rat liver rough endoplasmic reticulum [33]. It is considered that such crosslinking has relatively little effect on the substrate structure or substrate recognition of the target transporter, because of the small size of the azide group. Therefore, the PICK method was devised as a transporter identification method based on the strategy in Scheme 1. To our knowledge, this is the first successful application of the SWATH method for the molecular identification of a functional protein. The PICK method combines the use of azide-modified transporter substrates with the SWATH proteomic technique that affords excellent coverage and accuracy, and also utilizes inhibitors to increase the specificity/accuracy of transporter identification. In this method, it is important to avoid non-specific binding as much as possible, which is achieved by using several different types of inhibitors in combination and by keeping the reaction time between the photoreactive substrate and the cell very short. In principle, within the transporter complex, the proteins that bind to the substrate are likely, and the surrounding subunits are less likely, to be easily identified. Because the labeling reaction with azide is not sufficiently specific, we also introduced an additional criterion for SWATH analysis: inhibition by FLV, an inhibitor of the H^+^/OC antiporter. AzPYR shows affinity for the PYR-binding pocket of the antiporter (Figure 2A), and UV irradiation causes irreversible reaction of the azide group with the antiporter (Figure 2B). SWATH-MS was also carried out for samples under control (neither AzPYR nor FLV), AzPYR, and AzPYR+FLV conditions, finally affording sixteen candidate proteins (Table 1) based on the criteria shown in Scheme 1.

In the first functional screening of candidate proteins generated by the PICK method, TCIRG1, TM7SF3 and LHFPL6 were selected, since knockdown of these proteins affected H^+^/OC antiporter substrate transport (Table 2). Noting that the L-type amino acid trans-porter is a complex of LAT1 (SLC7A5) and 4F2hc (SLC3A2/CD98) [34], we hypothesized that two or all three candidates might be involved in H^+^/OC antiporter activity. It has been reported that TM7SF3, with seven putative transmembrane domains, inhibits the cytokine-induced death of pancreatic beta cells and promotes their insulin secretion [35]. TM7SF3 is a downstream transcriptional target of p53/TP53, and acts as a pro-survival homeostatic factor that attenuates the development of ER stress [36]. In addition, LHFPL6, with three putative transmembrane domains, is a candidate prognostic biomarker and therapeutic target for gastric cancer [37]. These two proteins have been reported to be expressed in diverse tissues and also in CNS cells [38,39,40,41,42,43]. Examination of the roles of these proteins in the CNS may provide important insights into the pharmacological effects of drugs and therapeutic strategies, but in the present work we chose to focus on the involvement of TM7SF3 and LHFPL6 in the H^+^/OC antiporter at the BBB, because the BBB protects the physiological function of the entire brain and significantly influences drug efficacy in the CNS. Interestingly, the mRNA expression levels of TM7SF3 and LHFPL6 in brain capillary endothelial cells are comparable to those of other transporters at the BBB (Figure S8) [44]. Indeed, in mice, TM7SF3 expression is greater than that of BCRP, which contributes to drug efflux at the BBB (Figure S8). In humans, LHFPL6 is more highly expressed than GLUT1 and LAT1 (Figure S8). Thus, TM7SF3 and LHFPL6 could contribute to transport at the BBB. TCIRG1 is a subunit of V-ATPase, which is responsible for acidifying and maintaining the pH of intracellular compartments in some cell types, and is also targeted to the plasma membrane, where it acidifies the extracellular environment [45]. In the second functional screening, the combination of TM7SF3 and LHFPL6 knockdown caused the greatest decrease in the uptake of H^+^/OC antiporter substrates, whereas TCIRG1 knockdown had a relatively weak effect (Table 3), strongly suggesting the involvement of TM7SF3 and LHFPL6 in the H^+^/OC antiporter activity.

In the gain-of-function analysis, the HEK293 expression system was used to investigate the contributions of TM7SF3 and LHFPL6 to the uptake of H^+^/OC antiporter substrates. Both transient and stable expression of TM7SF3 tended to increase the substrate uptake, whereas the expression of LHFPL6 had little impact (Figure 3 and Figure 4). In particular, the expression of TM7SF3 and LHFPL6 in HEK293 cells had little effect on the initial uptake. However, an increase in initial uptake of VAR, a relatively hydrophilic substrate, by TM7SF3-expressing cells (Figure 4) was observed.

Drug permeation through biological membranes is affected by the unstirred water layer surrounding the membrane, the plasma membrane, and intracellular binding. Therefore, to detect phenomena on the plasma membrane where the H^+^/OC antiporter is present, it is important to use a compound for which plasma membrane permeability is rate-limiting. Here, we synthesized a new compound, pyrilamine analogue (Figure 5 and Figure S4). The initial uptake rate of pyrilamine analogue was calculated to be 9.71 μL/mg protein/min, which was approximately 50-fold slower than PYR (Figure S9), implying that the plasma membrane permeability is rate-limiting in the initial uptake rate. In addition, it was proven in the present study to contain enough characteristics as a substrate of the H^+^/OC antiporter (Figure 6). This suggests that pyrilamine analogue would be suitable for clarifying the function of TM7SF3 and LHFPL6. Indeed, its uptake was significantly reduced by knockdown of TM7SF3 and/or LHFPL6 at any time from 0.5 to 30 min (Figure 7A). On the other hand, TM7SF3 and LHFPL6 knockdown did not affect gabapentin uptake (a substrate of LAT1) (Figure S10). Furthermore, the uptake of the pyrilamine analogue showed a 3.2-fold increase at 0.5 min in TM7SF3-stably expressing HEK293 cells (Figure 7C). All of these results suggest that TM7SF3 and LHFPL6 could be components of the H^+^/OC antiporter.

## 5. Conclusions

We developed the PICK method, which combines photo-affinity labeling and comprehensive proteomic analysis using SWATH-MS, to identify the molecular components of the H^+^/OC antiporter, which is responsible for the transport of various CNS drugs at the BBB. Sixteen candidate proteins were picked up based on predefined criteria. The results of knockdown and inhibitor studies in hCMEC/D3 cells, as well as uptake studies with overexpressing cells, indicated that TM7SF3 and LHFPL6 are H^+^/OC antiporter components. This information is expected to promote the development of effective CNS drugs and novel drug delivery systems. We anticipate that the PICK method will be useful for the identification of various transporters. When screening to identify the responsible transporters for compounds of interest, researchers frequently focus on known ABC, SLC and MFS transporters, and consequently may miss transporters that do not belong to these families. Furthermore, transporter complexes consisting of multiple proteins are difficult to identify. The PICK method can overcome these limitations and is expected to accelerate transporter discovery.

## Figures and Tables

**Table 1:** Sixteen proteins selected as H^+^/OC antiporter candidates.

Protein Names	Subcellular Location	Abundance Ratio AzPYR-Unbound Peptide			Number of Transmembrane Domains
		AzPYR /Control	(AzPYR+FLV) /Control	(AzPYR+DPH) /Control	
Magnesium transporter protein 1 (MAGT1)	Cell membrane	0.385	0.626	0.499	4
CD9 antigen (CD9)	Cell membrane	0.182	0.957	1.566	4
Cytochrome b reductase 1 (CYBRD1)	Unknown	0.401	0.975	0.925	6
Sodium/potassium-transporting ATPase subunit alpha-1 (ATP1A1)	Cell membrane	0.365	0.809	1.075	10
Cleft lip and palate transmembrane protein 1 (CLPTM1)	Cell membrane	0.266	0.658	0.692	5
V-type proton ATPase 116 kDa subunit a isoform 3 (TCIRG1)	Unknown	0.144	0.84	0.551	8
V-type proton ATPase 116 kDa subunit a isoform 1 (ATP6V0A1)	Unknown	0.319	0.739	― ^(a)^	8
LHFPL tetraspan subfamily member 6 protein (LHFPL6)	Unknown	0.0326	0.993	0.879	3
PRA1 family protein 3 (ARL6IP5)	Cell membrane	0.0891	0.0732	0.404	4
Solute carrier family 43 member 3 (SLC43A3)	Unknown	0.0725	0.0795	1	12
Transmembrane protein 65 (TMEM65)	Cell membrane	0.28	0.622	0.238	3
Transmembrane 7 superfamily member 3 (TM7SF3)	Cell membrane	0.451	0.681	― ^(a)^	7
Sodium/hydrogen exchanger 1 (SLC9A1)	Cell membrane	0.442	1.44	1.09	12
Solute carrier family 12 member 5 (SLC12A5)	Unknown	0.189	0.964	0.977	12
CD63 antigen (CD63)	Cell membrane	0.0906	0.172	0.413	4
Aquaporin-3 (AQP3)	Cell membrane	0.0561	0.494	0.0525	6

SWATH analysis was performed using the membrane fractions of hCMEC/D3 cells treated under the following four conditions: A, neither AzPYR nor inhibitor (control); B, AzPYR without inhibitor (AzPYR); C, AzPYR with FLV (AzPYR+FLV); D, AzPYR with DPH (AzPYR+DPH). The 16 proteins that were narrowed down through steps 1 to 6 (Scheme 1) are listed in this table. The subcellular location is taken from the Uniprot database. Abundance ratio for AzPYR-unbound peptide was calculated using the peptide peak area in SWATH analysis. The number of transmembrane domains was taken from the Uniprot database. AzPYR, azide-pyrilamine; FLV, fluvoxamine; DPH, diphenhydramine. Note that the experimental condition “AzPYR+DPH” was not used due to high cellular toxicity. ^(a)^ Values were not calculated due to poor data accuracy.

**Table 2:** First functional screening by single gene knockdown.

Target Gene	Relative Uptake (% of Control)			
	Pyrilamine	Tramadol	Diphenhydramine	Gabapentin
Control	100 ± 12	100 ± 10	100 ± 15	100 ± 7
MAGT1	109 ± 11	104 ± 8	118 ± 11	110 ± 5
CD9	26.0 ± 7.3	56.5 ± 7.1	83.4 ± 12.0	148 ± 17
CYBRD1	96.7 ± 19.0	114 ± 12	121 ± 23	165 ± 22 **
ATP1A1	130 ± 21	129 ± 22	132 ± 12	161 ± 17 **
CLPTM1	128 ± 19	133 ± 17	132 ± 7	169 ± 14 **
TCIRG1	50.2 ± 14.6	67.0 ± 11.7	83.9 ± 9.7	119 ± 5
SLC12A5	95.9 ± 7.6	87.2 ± 5.3	107 ± 9	92.8 ± 5.6
LHFPL6	46.1 ± 20.3	74.8 ± 13.9	68.9 ± 9.2	134 ± 14
ARL6IP5	174 ± 16	149 ± 2	134 ± 5	146 ± 14
SLC43A3	52.0 ± 16.0	122 ± 13	127 ± 21	143 ± 9
TMEM65	120 ± 19	170 ± 17 **	144 ± 16	130 ± 6
TM7SF3	69.5 ± 11.4	76.8 ± 5.5	68.7 ± 8.7	145 ± 8
SLC9A1	68.4 ± 16.3	76.8 ± 2.3	96.5 ± 8.7	87.3 ± 8.6
ATP6V0A1	116 ± 15	119 ± 3	107 ± 9	118 ± 7
CD63	105 ± 6	118 ± 8	97.7 ± 20.2	144 ± 14
AQP3	148 ± 45	114 ± 21	146 ± 25	90.8 ± 11.3

The uptakes of H^+^/OC antiporter substrates (pyrilamine, tramadol, and diphenhydramine) and a non-substrate (gabapentin) were assessed at 37 °C for 1 min in siRNA-transduced hCMEC/D3 cells. Each value represents the mean ± standard error (*n* = 4). ** *p* < 0.01, significantly different from the control.

**Table 3:** Second functional screening by multiple gene knockdown.

	Relative Uptake (% of Control)				
	Pyrilamine	Tramadol	Oxycodone	Varenicline	Gabapentin
Control	100 ± 9	100 ± 6	100 ± 7	100 ± 14	100 ± 5
TCIRG1 + LHFPL6	104 ± 2	112 ± 4	74.6 ± 1.8 *	61.0 ± 8.0	89.5 ± 6.1
TM7SF3 + LHFPL6	68.2 ± 5.7 *	64.8 ± 4.3 **	66.6 ± 3.0 **	67.7 ± 7.9	121 ± 6
TCIRG1 + TM7SF3	87.6 ± 9.0	93.0 ± 6.5	74.6 ± 7.6 *	59.6 ± 13.6	99.7 ± 6.9
Control	100 ± 4	100 ± 5	100 ± 6	100 ± 24	100 ± 3
TCIRG1 + TM7SF3 + LHFPL6	93.6 ± 5.5	101 ± 4	92.0 ± 4.1	76.0 ± 8.0	122 ± 5 *

The uptakes of H^+^/OC antiporter substrates (pyrilamine, tramadol, oxycodone, and varenicline) and a non-substrate (gabapentin) were evaluated at 37 °C for 1 min in hCMEC/D3 cells transduced siRNA for multiple targets. Each value represents the mean ± standard error (*n* = 4). * *p* < 0.05 and ** *p* < 0.01, significantly different from the control.

## Data Availability

The data of this study are available from the corresponding author upon reasonable request.

## Supplementary Materials

The following supporting information can be downloaded at: https://www.mdpi.com/article/10.3390/pharmaceutics14081683/s1, Detailed experimental methods, composition of media and buffers, and additional data are provided in the Supplementary Materials. Table S1: MRM transitions of test compounds, Table S2: siRNA and negative control siRNA information, Table S3: Sequences of sense and antisense primers (5′ to 3′) used for qPCR, Figure S1: Synthetic route of AzPYR from compound **1**, Figure S2: Detailed scheme of photoaffinity labeling for H^+^/OC antiporter using hCMEC/D3 cells, Figure S3: The detailed workflow for the SWATH analysis (step [3] in Scheme 1), Figure S4: Information on each compound in the synthetic route of pyrilamine analogue, Figure S5: mRNA expression in functional screening after single (A) or multiple (B) knockdown, Figure S6: Alteration of cell morphology by CD9 siRNA, Figure S7: Antipyrine uptake by HEK293 cells stably double-transfected with LHFPL6 and TM7SF3, Figure S8: Reported mRNA expression levels of TM7SF3, LHFPL6 and BBB transporters in human and mouse brain endothelial cells, Figure S9: Uptake study of pyrilamine in hCMEC/D3 cells, Figure S10: Uptake of gabapentin in hCMEC/D3 cells transfected with siRNA.

## References

[1] Yamazaki M., Terasaki T., Yoshioka K., Nagata O., Kato H., Ito Y., Tsuji A. (1994). Carrier-mediated transport of H1-antagonist at the blood-brain barrier: A common transport system of H1-antagonists and lipophilic basic drugs. Pharm. Res..

[2] Sadiq M.W., Borgs A., Okura T., Shimomura K., Kato S., Deguchi Y., Jansson B., Björkman S., Terasaki T., Hammarlund-Udenaes M. (2011). Diphenhydramine active uptake at the blood-brain barrier and its interaction with oxycodone in vitro and in vivo. J. Pharm. Sci..

[3] Boström E., Simonsson U.S., Hammarlund-Udenaes M. (2006). In vivo blood-brain barrier transport of oxycodone in the rat: Indications for active influx and implications for pharmacokinetics/pharmacodynamics. Drug Metab. Dispos..

[4] Cisternino S., Chapy H., André P., Smirnova M., Debray M., Scherrmann J.M. (2013). Coexistence of passive and proton antiporter-mediated processes in nicotine transport at the mouse blood-brain barrier. AAPS J..

[5] André P., Debray M., Scherrmann J.M., Cisternino S. (2009). Clonidine transport at the mouse blood-brain barrier by a new H^+^ antiporter that interacts with addictive drugs. J. Cereb. Blood Flow Metab..

[6] Kubo Y., Kusagawa Y., Tachikawa M., Akanuma S., Hosoya K. (2013). Involvement of a novel organic cation transporter in verapamil transport across the inner blood-retinal barrier. Pharm. Res..

[7] Tega Y., Akanuma S., Kubo Y., Hosoya K. (2015). Involvement of the H^+^/organic cation antiporter in nicotine transport in rat liver. Drug Metab. Dispos..

[8] Shimomura K., Okura T., Kato S., Couraud P.O., Schermann J.M., Terasaki T., Deguchi Y. (2013). Functional expression of a proton-coupled organic cation (H^+^/OC) antiporter in human brain capillary endothelial cell line hCMEC/D3, a human blood-brain barrier model. Fluids Barriers CNS.

[9] Okura T., Hattori A., Takano Y., Sato T., Hammarlund-Udenaes M., Terasaki T., Deguchi Y. (2008). Involvement of the pyrilamine transporter, a putative organic cation transporter, in blood-brain barrier transport of oxycodone. Drug Metab. Dispos..

[10] Shinozaki Y., Akanuma S., Mori Y., Kubo Y., Hosoya K. (2021). Comprehensive Evidence of Carrier-Mediated Distribution of Amantadine to the Retina across the Blood-Retinal Barrier in Rats. Pharmaceutics.

[11] Chapy H., Goracci L., Vayer P., Parmentier Y., Carrupt P.A., Declèves X., Scherrmann J.M., Cisternino S., Cruciani G. (2015). Pharmacophore-based discovery of inhibitors of a novel drug/proton antiporter in human brain endothelial hCMEC/D3 cell line. Br. J. Pharmacol..

[12] Hiranaka S., Tega Y., Higuchi K., Kurosawa T., Deguchi Y., Arata M., Ito A., Yoshida M., Nagaoka Y., Sumiyoshi T. (2018). Design, Synthesis, and Blood-Brain Barrier Transport Study of Pyrilamine Derivatives as Histone Deacetylase Inhibitors. ACS Med. Chem. Lett..

[13] Wang X., Qi B., Su H., Li J., Sun X., He Q., Fu Y., Zhang Z. (2017). Pyrilamine-sensitive proton-coupled organic cation (H^+^/OC) antiporter for brain-specific drug delivery. J. Control. Release.

[14] Okura T., Higuchi K., Kitamura A., Deguchi Y. (2014). Proton-coupled organic cation antiporter-mediated uptake of apomorphine enantiomers in human brain capillary endothelial cell line hCMEC/D3. Biol. Pharm. Bull..

[15] Kitamura A., Higuchi K., Okura T., Deguchi Y. (2014). Transport characteristics of tramadol in the blood-brain barrier. J. Pharm. Sci..

[16] Higuchi K., Kitamura A., Okura T., Deguchi Y. (2015). Memantine transport by a proton-coupled organic cation antiporter in hCMEC/D3 cells, an in vitro human blood-brain barrier model. Drug Metab. Pharmacokinet..

[17] Kurosawa T., Higuchi K., Okura T., Kobayashi K., Kusuhara H., Deguchi Y. (2017). Involvement of Proton-Coupled Organic Cation Antiporter in Varenicline Transport at Blood-Brain Barrier of Rats and in Human Brain Capillary Endothelial Cells. J. Pharm. Sci..

[18] Sweet D.H. (2021). Organic Cation Transporter Expression and Function in the CNS. Handb. Exp. Pharmacol..

[19] Alexovič M., Urban P.L., Tabani H., Sabo J. (2020). Recent advances in robotic protein sample preparation for clinical analysis and other biomedical applications. Clin. Chim. Acta.

[20] Frei A.P., Moest H., Novy K., Wollscheid B. (2013). Ligand-based receptor identification on living cells and tissues using TRICEPS. Nat. Protoc..

[21] Sumranjit J., Chung S.J. (2013). Recent advances in target characterization and identification by photoaffinity probes. Molecules.

[22] Patterson D.M., Nazarova L.A., Prescher J.A. (2014). Finding the right (bioorthogonal) chemistry. ACS Chem. Biol..

[23] Kitamura M., Yano M., Tashiro N., Miyagawa S., Sando M., Okauchi T. (2011). Direct Synthesis of Organic Azides from Primary Amines with 2-Azido-1,3-dimethylimidazolinium Hexafluorophosphate. Eur. J. Org. Chem..

[24] Tezuka K., Suzuki M., Sato R., Kawarada S., Terasaki T., Uchida Y. (2022). Activation of Annexin A2 signaling at the blood-brain barrier in a mouse model of multiple sclerosis. J. Neurochem..

[25] Uchida Y., Sasaki H., Terasaki T. (2020). Establishment and validation of highly accurate formalin-fixed paraffin-embedded quantitative proteomics by heat-compatible pressure cycling technology using phase-transfer surfactant and SWATH-MS. Sci. Rep..

[26] Sato R., Ohmori K., Umetsu M., Takao M., Tano M., Grant G., Porter B., Bet A., Terasaki T., Uchida Y. (2021). An Atlas of the Quantitative Protein Expression of Anti-Epileptic-Drug Transporters, Metabolizing Enzymes and Tight Junctions at the Blood-Brain Barrier in Epileptic Patients. Pharmaceutics.

[27] Uchida Y., Higuchi T., Shirota M., Kagami S., Saigusa D., Koshiba S., Yasuda J., Tamiya G., Kuriyama S., Kinoshita K. (2021). Identification and Validation of Combination Plasma Biomarker of Afamin, Fibronectin and Sex Hormone-Binding Globulin to Predict Pre-eclampsia. Biol. Pharm. Bull..

[28] Sadek B., Alisch R., Buschauer A., Elz S. (2013). Synthesis and dual histamine H_1_ and H_2_ receptor antagonist activity of cyanoguanidine derivatives. Molecules.

[29] Takamuro I., Sekine Y., Tsuboi Y., Nogi K., Taniguchi H. (2004). A Pyrazolopyrimidine Compound and a Process for Preparing the Same.

[30] Yamazaki M., Fukuoka H., Nagata O., Kato H., Ito Y., Terasaki T., Tsuji A. (1994). Transport mechanism of an H_1_-antagonist at the blood-brain barrier: Transport mechanism of mepyramine using the carotid injection technique. Biol. Pharm. Bull..

[31] Nakazawa Y., Okura T., Shimomura K., Terasaki T., Deguchi Y. (2010). Drug-drug interaction between oxycodone and adjuvant analgesics in blood-brain barrier transport and antinociceptive effect. J. Pharm. Sci..

[32] Hosang M., Gibbs E.M., Diedrich D.F., Semenza G. (1981). Photoaffinity labeling and identification of (a component of) the small-intestinal Na^+^,D-glucose transporter using 4-azidophlorizin. FEBS Lett..

[33] Kim S.H., Shin S.J., Park J.S. (1996). Identification of the ATP transporter of rat liver rough endoplasmic reticulum via photoaffinity labeling and partial purification. Biochemistry.

[34] Fotiadis D., Kanai Y., Palacín M. (2013). The SLC3 and SLC7 families of amino acid transporters. Mol. Asp. Med..

[35] Beck A., Isaac R., Lavelin I., Hart Y., Volberg T., Shatz-Azoulay H., Geiger B., Zick Y. (2011). An siRNA screen identifies transmembrane 7 superfamily member 3 (TM7SF3), a seven transmembrane orphan receptor, as an inhibitor of cytokine-induced death of pancreatic beta cells. Diabetologia.

[36] Isaac R., Goldstein I., Furth N., Zilber N., Streim S., Boura-Halfon S., Elhanany E., Rotter V., Oren M., Zick Y. (2017). TM7SF3, a novel p53-regulated homeostatic factor, attenuates cellular stress and the subsequent induction of the unfolded protein response. Cell Death Differ..

[37] Liu Y.J., Yin S.Y., Zeng S.H., Hu Y.D., Wang M.Q., Huang P., Li J.P. (2021). Prognostic Value of LHFPL Tetraspan Subfamily Member 6 (LHFPL6) in Gastric Cancer: A Study Based on Bioinformatics Analysis and Experimental Validation. Pharmgenomics Pers. Med..

[38] Whillans D.W., Adams G.E. (1975). Electron transfer oxidation of DNA radicals by paranitroacetophenone. Int. J. Radiat. Biol. Relat. Stud. Phys. Chem. Med..

[39] Petit M.M., Schoenmakers E.F., Huysmans C., Geurts J.M., Mandahl N., Van de Ven W.J. (1999). LHFP, a novel translocation partner gene of HMGIC in a lipoma, is a member of a new family of LHFP-like genes. Genomics.

[40] Hirano S., Goto R., Uchida Y. (2022). SWATH-Based Comprehensive Determination of the Localization of Apical and Basolateral Membrane Proteins Using Mouse Liver as a Model Tissue. Biomedicines.

[41] Usoskin D., Furlan A., Islam S., Abdo H., Lönnerberg P., Lou D., Hjerling-Leffler J., Haeggström J., Kharchenko O., Kharchenko P.V. (2015). Unbiased classification of sensory neuron types by large-scale single-cell RNA sequencing. Nat. Neurosci..

[42] Hodge R.D., Bakken T.E., Miller J.A., Smith K.A., Barkan E.R., Graybuck L.T., Close J.L., Long B., Johansen N., Penn O. (2019). Conserved cell types with divergent features in human versus mouse cortex. Nature.

[43] Smajić S., Prada-Medina C.A., Landoulsi Z., Ghelfi J., Delcambre S., Dietrich C., Jarazo J., Henck J., Balachandran S., Pachchek S. (2022). Single-cell sequencing of human midbrain reveals glial activation and a Parkinson-specific neuronal state. Brain.

[44] Yang A.C., Vest R.T., Kern F., Lee D.P., Agam M., Maat C.A., Losada P.M., Chen M.B., Schaum N., Khoury N. (2022). A human brain vascular atlas reveals diverse mediators of Alzheimer’s risk. Nature.

[45] Bronckers A.L., Lyaruu D.M., Bervoets T.J., Medina J.F., DenBesten P., Richter J., Everts V. (2012). Murine ameloblasts are immunonegative for Tcirg1, the v-H-ATPase subunit essential for the osteoclast plasma proton pump. Bone.

