# Supplementary material for: Proteomics-Based Transporter Identification by the PICK Method: Involvement of TM7SF3 and LHFPL6 in Proton-Coupled Organic Cation Antiport at the Blood–Brain Barrier
Source: Pharmaceutics. 2022 Aug 12;14(8):1683. doi: 10.3390/pharmaceutics14081683 (PMC9413594; doi:10.3390/pharmaceutics14081683)
Supplement: Supplementary file 1 [file pharmaceutics-14-01683-s001.zip › pharmaceutics-1804041-supplementary.pdf]

Supplementary Materials

Buffer contents

・Uptake buffer;

122 mM NaCl, 3 mM KCl, 25 mM $NaHCO_3$, 1.2 mM $MgSO_4$, 1.4 mM $CaCl_2$, 10 mM D-glucose, 10 mM HEPES, pH 7.4

・Glucose-free buffer;

122 mM NaCl, 3 mM KCl, 25 mM $NaHCO_3$, 1.2 mM $MgSO_4$, 1.4 mM $CaCl_2$, 10 mM 3-O-methylglucose, 10 mM HEPES, 0.1% NaN_3, pH 7.4

・Na^+-free buffer;

122 mM LiCl, 3 mM KCl, 25 mM $KHCO_3$, 1.2 mM $MgSO_4$, 1.4 mM $CaCl_2$, 10 mM D-glucose, 10 mM HEPES, pH 7.4

or

122 mM cholineCl, 3 mM KCl, 25 mM $KHCO_3$, 1.2 mM $MgSO_4$, 1.4 mM $CaCl_2$, 10 mM D-glucose, 10 mM HEPES, pH 7.4

・Membrane potential-free buffer;

125 mM KCl, 25 mM $KHCO_3$, 1.2 mM $MgSO_4$, 1.4 mM $CaCl_2$, 10 mM D-glucose, 10 mM HEPES, pH 7.4

Cell culture conditions

hCMEC/D3 is a human brain capillary endothelial cell line immortalized by lentiviral transduction of the catalytic subunit of human telomerase and SV40 T antigen. hCMEC/D3 cells were cultured in EBM 2 medium (Lonza, Basel, Switzerland) supplemented with fetal bovine serum, growth factors (VEGF, R3 IGF, hEGF, bFGF and hydrocortisone), penicillin, streptomycin and HEPES on

collagen-coated dishes. For uptake experiments, hCMEC/D3 cells between 25 and 35 passages were seeded on rat collagen I-coated 24-well plates (BD Bioscience, San Jose, CA, USA) at a density of 4.0×10^4 cells/cm^2. Uptake was evaluated after the cells reached confluence. The cells were washed with transport buffer and preincubated with the buffer for 20 min at 37 °C or 4 °C. After preincubation, the transport buffer containing drugs and compounds was added to each well. The cells were incubated at 37 °C or 4 °C for a designated time, and then washed three times with ice-cold transport buffer to terminate the uptake. The cells were collected with a scraper in 200 μL of H_2O, and stored in a freezer set at −30 °C until analysis. Cellular protein content was determined with a Micro BCA protein assay kit (Thermo Fisher Scientific Inc., Waltham, MA, USA).

To estimate kinetic parameters from the in vitro uptake results, the uptake data were fitted to the equation in the text by nonlinear least-squares regression analysis using Prism software (Graphpad, San Diego, CA, USA). In the equation, V is the initial uptake velocity of pyrilamine analogue, V_{max} is the maximum uptake rate, S is the concentration in the uptake buffer, K_m is the Michaelis constant, and K_d is non-saturable uptake clearance.

LC-MS/MS analysis

The test drugs were quantified using a HPLC-tandem mass spectrometry system composed of a Nexera-XR (Shimazu, Kyoto, Japan) HPLC system connected to a Qtrap4500 (AB Sciex, Foster City, CA, USA) mass spectrometer with an electrospray ionization interface. Samples were diluted with methanol and the mobile phase containing the internal standard alprenolol. Chromatographic separation was achieved on a XR-ODS (2.0 × 30 mm, 2.2 μm, Shimazu, Kyoto, Japan) for substrate drugs for H^+/OC antiporter and Synergi Hydro-RP column (2.0 × 50 mm, 2.5 μm; Phenomenex, Torrance, California) for pyrilamine analogue. The mobile phase for gradient analysis was composed of solvent A (10 mM ammonium formate buffer containing 0.2% formate) and solvent B (methanol).

The LC gradient using XR-ODS was programmed as follows; 0-0.2 min 0% B, 0.2-1.3 min linear increase to 99%, kept constant to 2.0 min, then the starting condition was restored and maintained for 0.6 min to equilibrate the system (flow rate 700 μL/min). The gradient using Hydro-RP was programmed as follows; 0–1 min 0% B, 1–2 min linear increase to 95% B, kept constant to 4 min, and finally the initial condition was restored and held for 1 min to re-equilibrate the system (flow rate 400 μL/min). The column temperature was set at 40°C. Analyst 1.6.1 software was used to collect data and to control the QTRAP4500 system. The MRM transitions of test compounds are shown in Supplementary Table S1.

Supplemental Table S1. MRM transitions of test compounds.

Test compound	MRM transition		DP (volts)	EP (volts)	CE (volts)	CXP (volts)
	Q1 mass	Q3 mass				
Alprenolol	250.2	116.0	70.0	11.0	22.8	14.0
Antipyrine	188.9	77.0	61.0	10.0	55.0	8.0
Diphenhydramine	256.1	167.0	40.0	10.0	25.0	15.0
Pyrilamine analogue	443.2	121.0	46.0	10.0	47.0	10.0
Gabapentin	172.0	154.0	60.0	10.0	17.0	13.0
Oxycodone	316.2	298.0	51.0	10.0	30.0	13.0
Pyrilamine	286.0	120.9	31.0	10.0	29.0	12.0
Tramadol	264.2	58.0	31.0	10.0	47.0	6.0
Varenicline	212.0	169.0	75.0	10.0	13.0	12.0

Supplemental Table S2. siRNA and negative control siRNA information.

Protein names	siRNA ID	
Magnesium transporter protein 1	s38424	s38425
CD9 antigen	s2598	s2599
Cytochrome b reductase 1	s36608	s230761
Sodium/potassium-transporting ATPase subunit alpha-1	s1720	s1718
Cleft lip and palate transmembrane protein 1	s3185	s3187
V-type proton ATPase 116 kDa subunit a isoform 3	s20159	s20158
V-type proton ATPase 116 kDa subunit a isoform 1	s1809	s1810
LHFPL tetraspan subfamily member 6 protein	s19849	s19847
PRA1 family protein 3	s20690	s223129
Solute carrier family 43 member 3	s26391	s223813
Transmembrane protein 65	s459575	s45958
Transmembrane 7 superfamily member 3	s28646	s28647
Sodium/hydrogen exchanger 1	s13021	s13022
Solute carrier family 12 member 5	s33088	s33089
CD63 antigen	s2699	s2701
Aquaporin-3	s1521	s1523
Negative control siRNA	Qiagen (Cat No. 1022076)	

Supplemental Table S3. Sequences of sense and antisense primers (5’ to 3’) used for qPCR.

Primer sequences	sense	anti-sense
Magnesium transporter protein 1	AGTTCCGTCGCCTTGTGAAA	CAGCTTGCTTGCAAACGACA
CD9 antigen	CTTCATCTTCTGGCTTGCCG	GCTTGAAGATGCTCTTGGTCT
Cytochrome b reductase 1	CTCGTCTGGGTCCTCCACTA	TGTAGACGATGATGGCGATGC
Sodium/potassium-transporting ATPase subunit alpha-1	TTGGGCACTGACATGGTTCC	TCAGCCGCTCATTCACAAGT
Cleft lip and palate transmembrane protein 1	GCTGCTATGCCGTCTACAGT	GGTGATGAAGCCGAAGGTCA
V-type proton ATPase 116 kDa subunit a isoform 3	AGTTCAGAGACCTCAACGCC	AGGTCTTCTCCAGCTCCTCA
V-type proton ATPase 116 kDa subunit a isoform 1	GAGGACGCAGACGAGTTTGA	CGCAAGTAGGAGGCAGTGTT
LHFPL tetraspan subfamily member 6 protein	CATGGGTTGCTGTGTTTCCG	ACCAATCAACAAGCCCCCAA
PRA1 family protein 3	TGGAACAACCGCGTAGTGAG	GGGACTCAGAAACCCCACAA
Solute carrier family 43 member 3	CTCAGCCGTGCTGCTCTTC	ATCTGCAGGTTGGTGATGAGAA
Transmembrane protein 65	CGCGACTTCATCTACAGCCT	AGCTTCCAATTTTTCTTGGGCA
Transmembrane 7 superfamily member 3	GGAATTCCAGCGAGGGTCTTA	ATAGCTTCCTCTGGAAAGGGC
Sodium/hydrogen exchanger 1	CCTGTTCCTCACTGCCATCA	CGCTTCGTCTCTTGCTTTTTC
Solute carrier family 12 member 5	TTCAGCATGAAGCCGGAGTG	TGAGCAAAACAAGCTTGGCG
CD63 antigen	TGGCCTTTTGCGCCTGT	AGCCCCCTGGATTATGGTCT
Aquaporin-3	ACCAGCTTTTTGTTTCGGGC	GGCTGTGCCTATGAACTGT
TATA-binding protein	TGCACAGGAGCCAAGAGTGAA	CACATCACAGCTCCCCACCA

Supplemental Figure S1. Synthetic route to AzPYR from compound **1**.

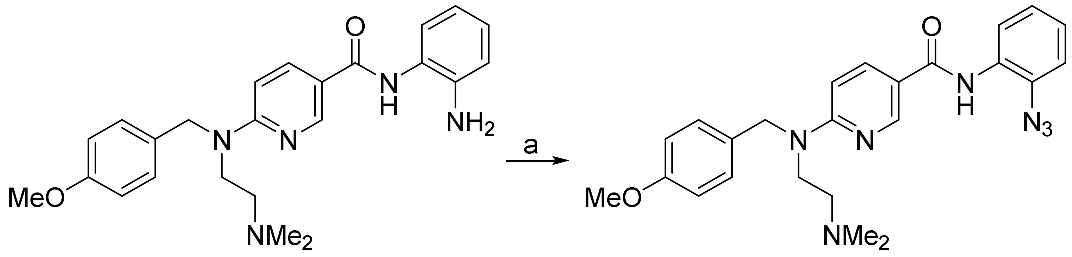

1 **AzPYR**

Reagents and condition: (a) 2-azido-1,3-dimethylimidazolinium hexafluorophosphate, DMAP, CH_2Cl_2, 66%

Supplemental Figure S2. Detailed scheme of photoaffinity labeling for H^+/OC antiporter using hCMEC/D3 cells.

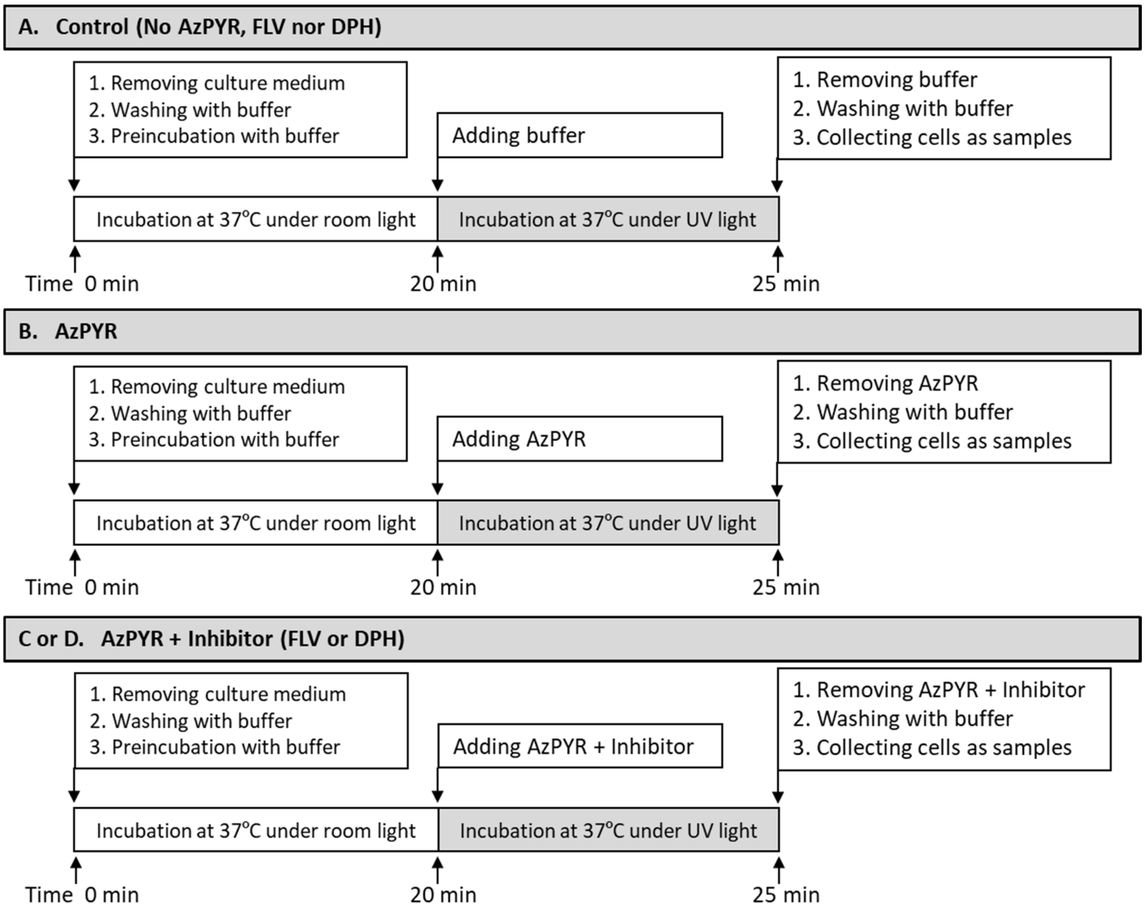

AzPYR; azide-pyrilamine, FLV; fluvoxamine, DPH; diphenhydramine.

Supplemental Figure S3. The detailed workflow for the SAWATH analysis (step [3] in Scheme 1).

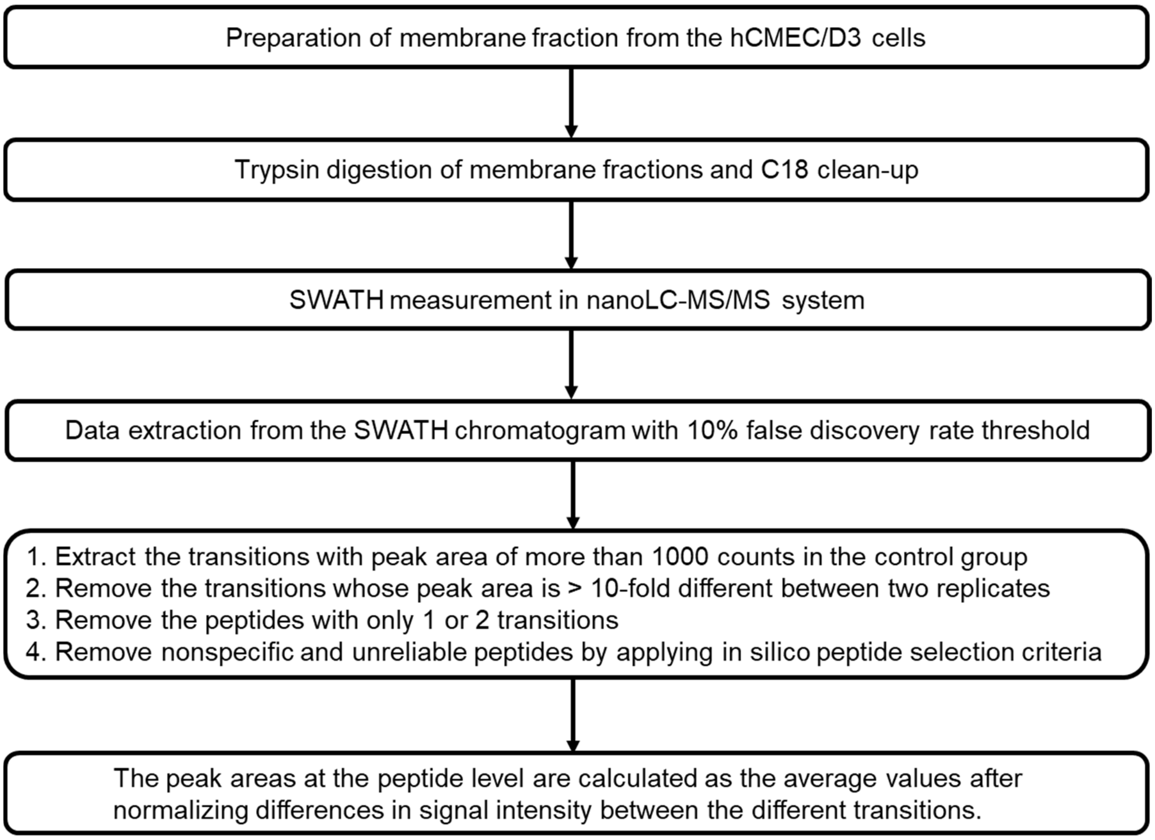

Supplemental Figure S4. Information on each compound in the synthetic pathway to the pyrilamine analogue.

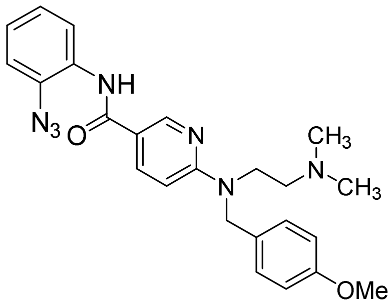

***N*-(2-Azidophenyl)-6-((2-(dimethylamino)ethyl)(4-methoxybenzyl)amino) nicotinamide (AzPYR)**

A mixture of compound **1** (30 mg), 2-azido-1,3-dimethylimidazolinium hexafluorophosphate (23 mg) and dimethylaminopyridine (9.6 mg) in CH_2Cl_2 (0.5 mL) was stirred at room temperature for 6 h. The reaction was quenched by addition of satd $NaHCO_3$ aq and the organic layer was separated. The water layer was extracted with AcOEt three times. The combined organic layer was washed with brine, dried over Na_2SO_4 and filtered. Concentration of the filtrate under reduced pressure of the organic layer and silica gel column chromatography (NH_2 silica gel, hexane/AcOEt = 1/1) of the crude product afforded the target product (AzPYR; 21 mg, 66%).

^{1}H NMR (400 MHz, $CDCl_3$) δ 2.28 (s, 6H), 2.52 (t, J = 7.3 Hz), 3.71 (t, J = 7.3 Hz), 3.79 (s, 3H), 4.79 (s, 2H), 6.53 (d, J = 8.8 Hz, 2H), 6.86 (d, J = 8.8 Hz, 2H), 7.11–7.21 (m, 5H), 7.93 (dd, J = 8.8, 2.4 Hz, 1H), 8.14 (s, 1H), 8.50 (dd, J = 8.0, 2.0 Hz, 1H), 8.74 (d, J = 2.4 Hz, 1H); ^{13}C NMR (100 MHz, $CDCl_3$) δ 45.8, 46.7, 51.5, 55.3, 56.6, 105.3, 114.1, 117.6, 118.1, 120.7, 124.1, 125.6, 127.8, 128.1, 129.5, 129.9, 136.6, 147.9, 158.8, 159.7, 164.0; HRMS (ESI) *m*/*z* calcd for $C_{24}H_{28}N_7O_2$ 446.2299 $(M+H)^+$, found 446.2298.

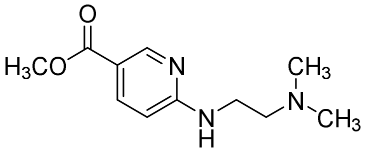

Methyl 6-[[2-(dimethylamino)ethyl]amino]-3-pyridinecarboxylate (2)

To a solution of methyl 6-bromonicotinate (**1**, 683 mg, 3.2 mmol) in pyridine (15 mL) was added *N*,*N*-dimethylethylendiamine (1.7 mL, 15.8 mmol). The mixture was refluxed at 140 °C for 3 h. After removal of the solvent, H_2O was added to the residue. The mixture was extracted with CH_2Cl_2, and dried over $MgSO_4$. After removal of the solvent, the residue was purified by column chromatography (silica gel, methanol/ethyl acetate = 1/2) to give **2** (640 mg, 91%) as a white solid: mp 30-32 °C: ^{1}H NMR (600 MHz, $CDCl_3$) δ 2.26 (s, 6H), 2.54 (t, J = 6.2 Hz, 2H), 3.39–3.42 (m, 2H), 3.85 (s, 3H), 5.59 (br, 1H), 6.36 (d, J = 8.9 Hz, 1H), 7.95 (dd, J = 2.1, 8.9 Hz, 1H), 8.74 (d, J = 2.1 Hz, 1H); ^{13}C NMR (150 MHz, $CDCl_3$) δ 38.9, 45.0, 51.5, 57.5, 114.8, 138.1, 151.5, 160.7, 166.5; IR (ATR) 2952, 1716, 1608 cm^{-1}; HRMS (ESI) *m*/*z* calcd for $C_{11}H_{17}N_3O_2$ 224.1394 $(M+H)^+$, found 224.1397.

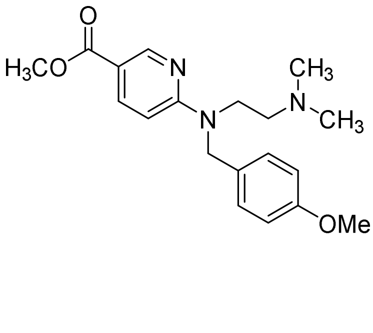

Methyl 6-{[2-(dimethylamino)ethyl][(4-methoxyphenyl)methyl]amino}pyridine-3-carboxylate (3)

To a solution of **2** (35 mg, 0.16 mmol) in THF (1.5 mL) was added sodium hydride (60% in oil) (9 mg, 0.23 mmol) at 0 °C under argon. The mixture was stirred for 30 min at 25 °C, cooled to 0 °C, and treated with 4-methoxybenzyl chloride (43 □l, 0.31 mmol). After being stirred overnight at 25 °C, the mixture was treated with H_2O, and extracted with Et_2O. The organic solution was dried, and after removal of the solvent, the residue was purified by column chromatography (silica gel, methanol/ethyl acetate = 1/10) to give **3** (19 mg, 34%) as a pale yellow oil.

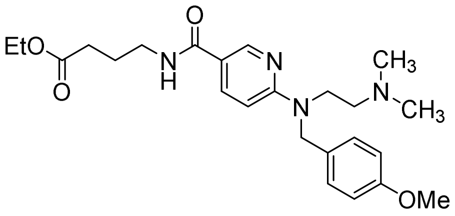

Ethyl 4-[[[6-[[2-(dimethylamino)ethyl]-[(4-methoxyphenyl)methyl]amino]-3-pyridinyl]carboxyl]amino] butanoate (4)

To a solution of **3** (192 mg, 0.56 mmol) in MeOH (5.6 mL) was added 6 N *aq*. NaOH (2.8 ml). The mixture was stirred at 100 °C for 30 min, neutralized with 1 N HCl, and evaporated. MeOH was added to the residue, and the mixture was filtered. The filtrate was evaporated in vacuo to give the crude carboxamide, which was used directly for the next step without further purification. To a solution of the carboxamide in DMF (5 ml) was added 1-hydroxybenzotriazole (83 mg, 0.62 mmol) and 1-ethyl-3-(3-dimethylaminopropyl)carbodiimide hydrochloride (118 mg, 0.62 mmol). The mixture was allowed to react at room temperature for 30 min, then ethyl 4-aminobutyrate hydrochloride (103 mg, 0.62 mmol) and *N*,*N*-diisopropylethylamine (190 □l, 1.11 mmol) were added. The mixture was stirred for 3.5 h at 25 °C, then water was added , and the aqueous solution was extracted with CH_2Cl_2. The combined organic layer was washed with brine, and dried over $MgSO_4$. After removal of the solvent, the residue was purified by column chromatography (silica gel, methanol/ethyl acetate = 1/5) to give **4** (184 mg, 74%) as a pale yellow oil: ^{1}H NMR (600 MHz, $CDCl_3$) δ 1.24 (t, J = 7.6 Hz, 3H), 1.93 (dd, J = 6.9, 6.9 Hz, 2H), 2.26 (s, 6H), 2.42 (t, J = 6.9 Hz, 2H), 2.49 (t, J = 7.6 Hz, 2H), 3.48 (dt, J = 5.5, 6.9 Hz, 2H), 3.67 (t, J = 7.6 Hz, 2H), 4.12 (q, J = 7.6 Hz, 2H), 4.74 (s, 2H), 6.33 (t, J = 5.5 Hz, 1H), 6.46 (d, J = 8.9 Hz, 1H), 6.83 (d, J = 8.9 Hz, 2H), 7.13 (d, J = 8.9 Hz, 2H), 7.82 (dd, J = 2.8, 8.9 Hz, 1H), 8.57 (d, J = 2.8 Hz, 1H); ^{13}C NMR (150 MHz, $CDCl_3$) δ 14.1, 24.5, 32.0, 39.4, 45.7, 46.6, 51.4, 55.2, 56.5, 60.6, 104.9, 114.0, 117.9, 128.1, 129.8, 136.4, 147.5, 158.7, 159.5, 166.2, 173.8; IR (ATR) 2936, 1729, 1600, 1501 cm^{-1}; HRMS (ESI) *m*/*z* calcd for $C_{24}H_{34}N_4O_4$ 443.2653 (M+H)$^+$, found 443.2654.

Supplemental Figure S5. mRNA expression in functional screening by single (A) or multiple (B) gene knockdown.

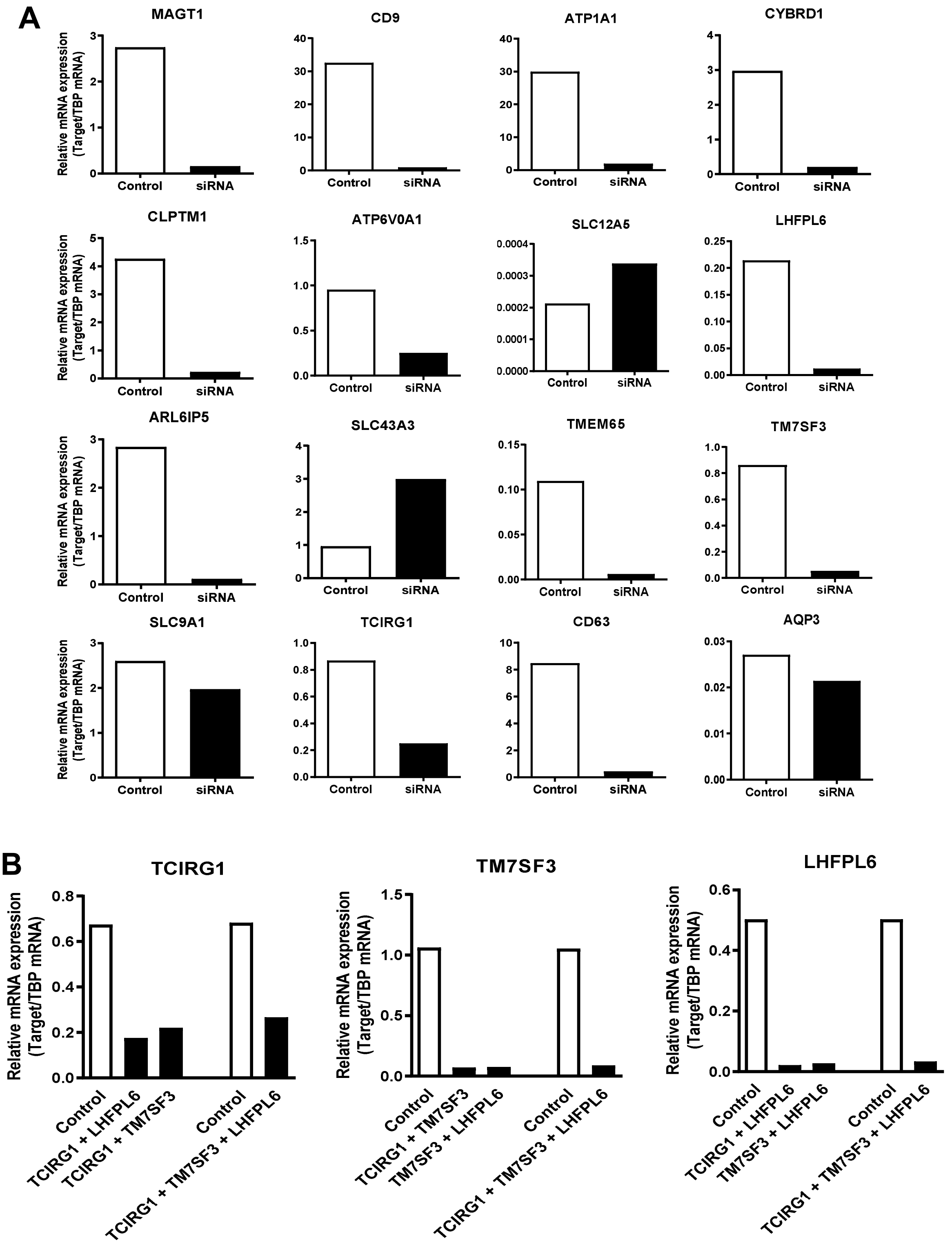

The mRNA was isolated from hCMEC/D3 cells 48 h after incubation with siRNA for 24 h. To increase screening throughput, the mRNA was quantified without biological replicates.

Supplemental Figure S6. Alteration of cell morphology by siRNA knockdown of CD9.

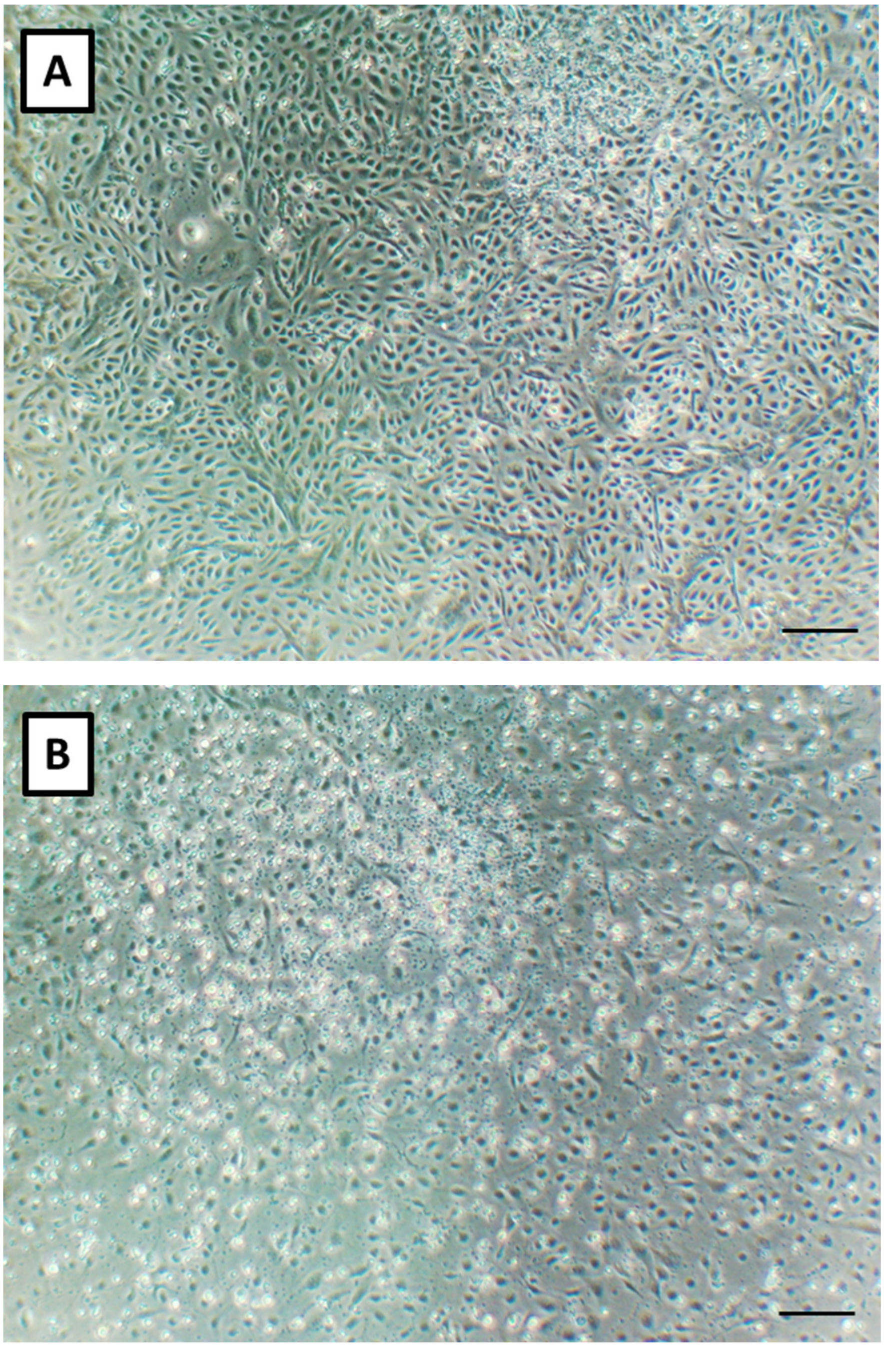

hCMEC/D3 cells after siRNA treatment for 48 hr (A; negative control, B; siRNA CD9). The scale bar is 100 µm.

Supplemental Figure S7. Antipyrine uptake by HEK293 cells stably double-transfected with LHFPL6 and TM7SF3.

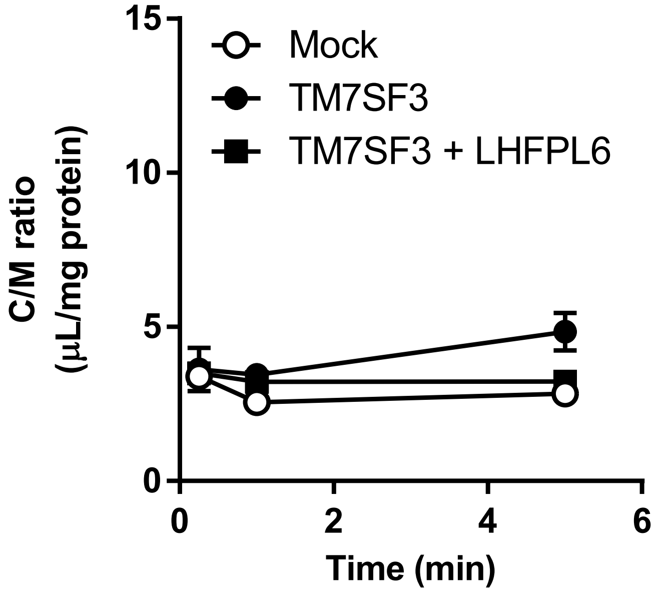

The time-course of antipyrine uptake was assessed at 37 °C in mock (closed circle) and HEK293 cells stably transfected with TM7SF3 (open circle) or both LHFPL6 and TM7SF3 (open square). Each point represents the mean ± standard error (n = 3).

Supplemental Figure S8. Reported mRNA expression levels of TM7SF3, LHFPL6 and BBB transporters in human and mouse brain endothelial cells.

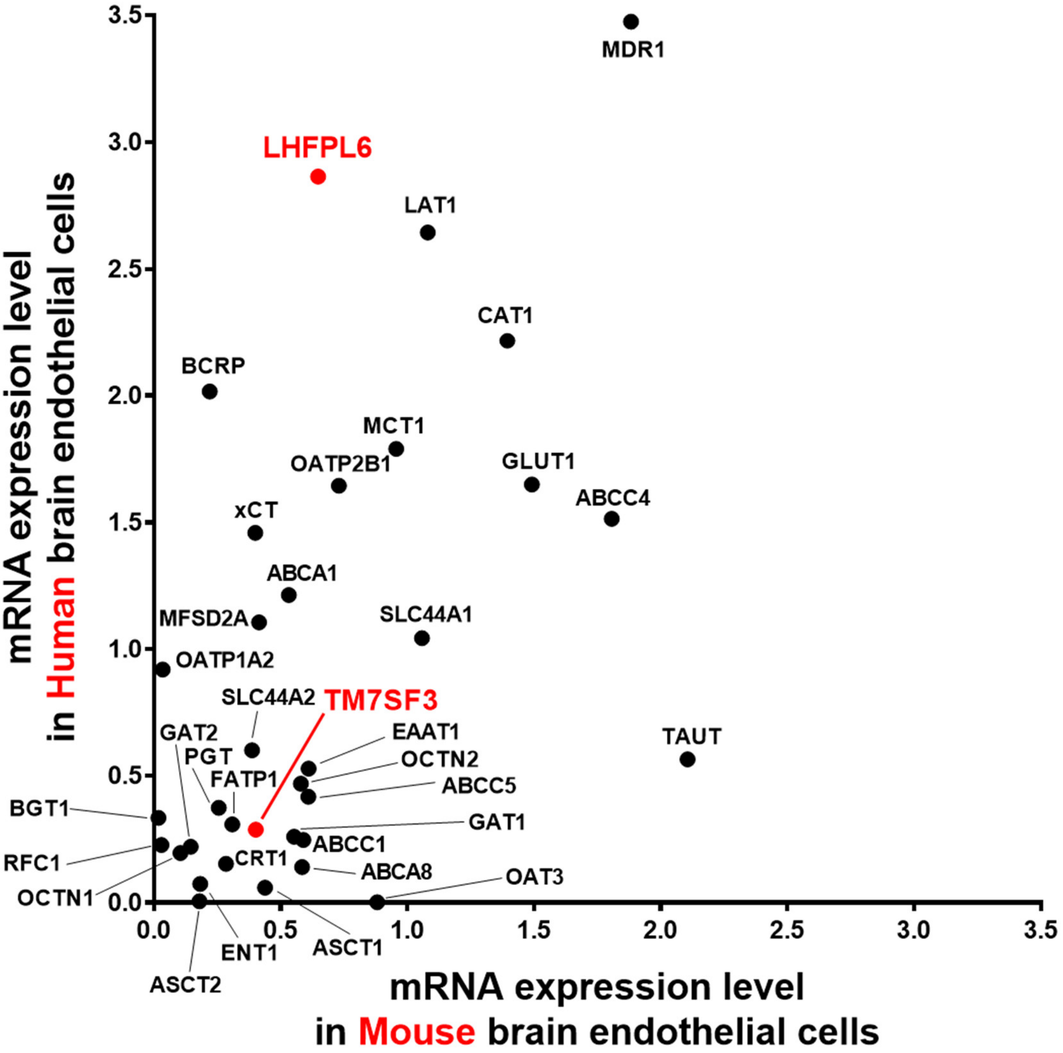

This figure was generated from transporter and TM7SF3 and LHFPL6 mRNA expression levels extracted from reported RNAseq data for human and mouse brain endothelial cells (Nature, 603, 885–892, 2022).

Supplemental Figure S9. Uptake study of pyrilamine in hCMEC/D3 cells.

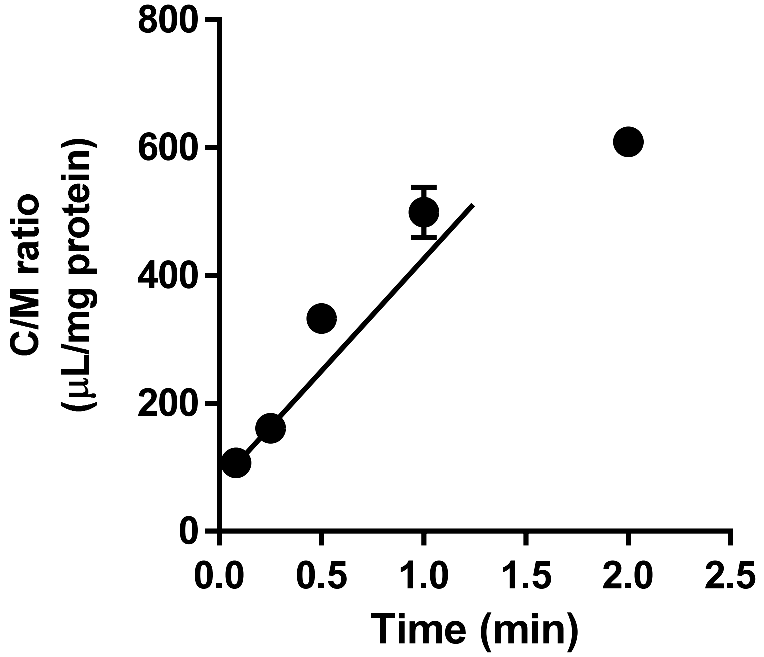

Time-dependent uptake of pyrilamine (0.5 μM) into hCMEC/D3 cells was evaluated at 37 °C at the designated times. Initial uptake clearance was calculated as the slope from 5 s to 1 min. Each value represents the mean ± standard error (n = 4).

Supplemental Figure S10. Uptake of gabapentin in hCMEC/D3 cells transfected with siRNA.

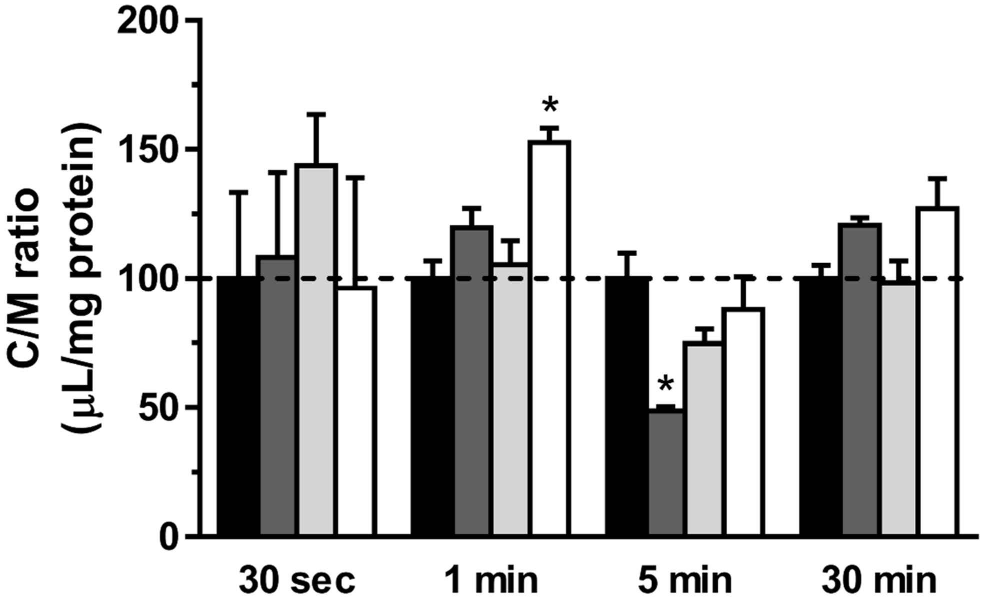

Uptake of gabapentin (10 μM) in hCMEC/D3 cells with knockdown of TM7SF3 and LHFPL6 was assessed by simultaneous addition of pyrilamine analogue. The black, dark gray, light gray and white columns represent negative control, TM7SF3 knockdown, LHFPL6 knockdown and double knockdown, respectively. Each value represents the mean ± standard error (n = 4). * $p < 0.05$ indicates a significant difference versus the negative control.
